# Motifs and *cis*-regulatory modules mediating the expression of genes co-expressed in presynaptic neurons

**DOI:** 10.1186/gb-2009-10-7-r72

**Published:** 2009-07-01

**Authors:** Rui Liu, Sridhar Hannenhalli, Maja Bucan

**Affiliations:** 1 Department of Genetics and Penn Center for Bioinformatics, University of Pennsylvania, Philadelphia, PA 19104, USA

## Abstract

**Background:**

Hundreds of proteins modulate neurotransmitter release and synaptic plasticity during neuronal development and in response to synaptic activity. The expression of genes in the pre- and post-synaptic neurons is under stringent spatio-temporal control, but the mechanism underlying the neuronal expression of these genes remains largely unknown.

**Results:**

Using unbiased *in vivo *and *in vitro *screens, we characterized the *cis *elements regulating the *Rab3A *gene, which is expressed abundantly in presynaptic neurons. A set of identified regulatory elements of the *Rab3A *gene corresponded to the defined *Rab3A *multi-species conserved elements. In order to identify clusters of enriched transcription factor binding sites, for example, *cis*-regulatory modules, we analyzed intergenic multi-species conserved elements in the vicinity of nine presynaptic genes, including *Rab3A*, that are highly and specifically expressed in brain regions. Sixteen transcription factor binding motifs were over-represented in these multi-species conserved elements. Based on a combined occurrence for these enriched motifs, multi-species conserved elements in the vicinity of 107 previously identified presynaptic genes were scored and ranked. We then experimentally validated the scoring strategy by showing that 12 of 16 (75%) high-scoring multi-species conserved elements functioned as neuronal enhancers in a cell-based assay.

**Conclusions:**

This work introduces an integrative strategy of comparative genomics, experimental, and computational approaches to reveal aspects of a regulatory network controlling neuronal-specific expression of genes in presynaptic neurons.

## Abstract 2

An integrative strategy of comparative genomics, experimental and computational approaches reveals aspects of a regulatory network controlling neuronal-specific expression in presynaptic neurons.

## Background

Synaptic transmission, the crucial process that enables information transfer in the nervous system, is a series of events in which neurotransmitters are released via exocytosis from presynaptic neurons and taken up by postsynaptic neurons. In presynaptic neurons, synaptic vesicles facilitate uptake of neurotransmitters and dock at the active zone of the plasma membrane. In response to calcium signaling, vesicles rapidly fuse with the plasma membrane and release neurotransmitters by exocytosis. The vesicles are recycled by subsequent endocytosis. These events are orchestrated by multiple protein complexes [1,2]. For example, one class of proteins is attached to the synaptic vesicle membrane, and is involved in calcium sensing (SYT1 and SV2a), membrane fusion (VAMP1), and vesicle recycling (SCAMP5). Another group of proteins is bound with scaffold proteins or directly anchored at the active zone and functions in vesicle docking (SYN1 and RIMs), priming (RIMs) and fusion (SNAP25 and STXBP1). In addition to these proteins, RAB3 proteins (RAB3A and RAB3C) function as molecular linkers between synaptic vesicles and the active zone by cycling between vesicle-associated and dissociated forms and interacting with multiple effectors, such as RIMs and SYN1 [3-5].

To ensure precisely controlled synaptic communication, members of protein complexes in presynaptic neurons have highly coordinated expression and protein localization [6-9]. Spatial and temporal expression patterns of several presynaptic genes have been reported in detail. For instance, in mammalian brain, *Rab3A *is expressed throughout all brain regions, including cortex, hippocampus, cerebellum and thalamus [10,11]. In the mouse, detectable levels of *Rab3A*, *Syp *and *Sv2a *mRNAs are reported from embryonic day 9.5 or 10.5, an early neurogenesis stage in which progenitors gradually undergo cell cycle withdrawal and neuronal differentiation [12-14]. During neuronal maturation and synapse formation, *Rab3A *expression dramatically increases and the protein becomes localized to the presynaptic terminal of neurons [15,16]. In contrast to the increased expression during neuronal development, neurodegenerative and psychiatric disorders such as Alzheimer's, Huntington's disease and schizophrenia are marked by decreased levels of RAB3A, SYT1, and SNAP25, coupled with the loss of functional synapses [17-19]. It is clear that both gene expression and protein distribution in presynaptic neurons are tightly regulated during neuronal development, differentiation and maintenance. However, *cis*-regulatory mechanisms mediating the neuronal expression of presynaptic genes still remain unknown.

Comparative genomics has taken advantage of the increasing number of whole genome sequences available for many model organisms in order to identify unknown regulatory elements [20-26]. For example, 353 of 868 multi-species conserved elements (MCEs) examined by *in vivo *enhancer assay using mouse transgenesis were associated with tissue-specific expression of the reporter gene [27,28]. Furthermore, investigations of the promoter regions of co-expressed genes have led to discovery of significant clusters of transcription factor binding sites, that is, *cis*-regulatory modules (CRMs) [29-33]. Although these common CRMs are statistically over-represented in regulatory elements of co-expressed genes, their arrangement and activity may vary greatly [34-36]. Therefore, co-expressed genes defined from large-scale expression studies provide excellent means to study common tissue-specific regulatory modules.

To elucidate the common CRMs regulating neuronal-specific expression of presynaptic genes, we first defined a cluster of nine presynaptic genes that were highly and specifically expressed in neuronal tissues. Unbiased *in vivo *and *in vitro *screens of *cis*-regulatory sites for *Rab3A*, one of the nine genes, revealed regulatory roles for all MCEs in the vicinity of this gene. We thus identified motifs of 16 transcription factors that were enriched in intergenic MCEs of the nine presynaptic genes in comparison to ubiquitously expressed genes. Identified CRMs were then used to develop a novel metric to rank MCEs according to their potential for mediating neuronal-specific expression. By experimentally validating the high-scoring as well as the low-scoring MCEs, we confirmed that this CRM-based scoring metric accurately identified neuronal-specific regulatory elements.

## Results

### Gene selection for neuronal-restricted expression

**Figure 1:**
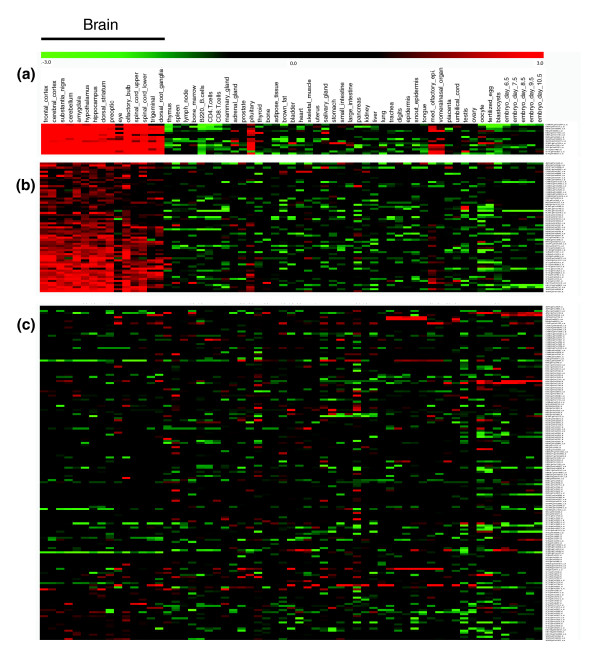
**Expression analysis of 107 presynaptic genes across mouse tissues and developmental stages**. Expression profile of 107 genes corresponding to 161 oligonucleotide gene probes were clustered into 3 distinct expression groups by the hierarchical k-means clustering method. **(a) **Cluster 1: transcripts with high expression in brain tissues and low levels of expression in other tissues. **(b) **Cluster 2: transcripts with moderate expression in brain tissues and low levels of expression in other tissues. **(c) **Cluster 3: transcripts with widespread but low levels of expression in most tested tissues.

In our previous work, we performed comparative sequence analysis of presynaptic genes encoding proteins mainly present in the presynaptic nerve terminal [37]. To select genes with neuronal-restricted expression, we used SymAtlas to examine the expression profiles of these genes obtained by microarray analysis in 54 mouse non-redundant tissues and at 7 developmental stages [38]. Expression profiles for 107 presynaptic genes, represented by 161 oligonucleotide gene probes with a consistent expression pattern, were available. A hierarchical k-means clustering of the 107 gene expression patterns revealed three distinct groups (Figure 1; Table S1 in Additional data file 1): nine genes, including *Rab3A*, *Rab3C*, *Scamp5*, *Snap25*, *Stxbp1*, *Syn1*, *Sv2a*, *Camk2N1 *and *Dnm1*, are tightly clustered with the most abundant expression restricted to brain tissues (cluster 1, Figure 1a, Table 1); 27 genes are grouped with moderate neuronal expression (cluster 2, Figure 1b); and 71 genes are expressed in all tested tissues with an ubiquitous or nonspecific pattern (cluster 3, Figure 1c).

**Table 1:** Clustering of nine presynaptic genes highly expressed in brain

#	Cluster	Gene	Brief description
1	1	*CAMK2N1*	Calcium/calmodulin-dependent protein kinase II inhibitor 1
2	1	*DNM1*	Dynamin 1
3	1	*RAB3A*	RAB3A, member RAS oncogene family
4	1	*RAB3C*	RAB3C, member RAS oncogene family
5	1	*SCAMP5*	Secretory carrier membrane protein 5
6	1	*SNAP25*	Synaptosomal-associated protein 25
7	1	*STXBP1*	Syntaxin binding protein 1
8	1	*SV2A*	Synaptic vesicle glycoprotein 2a
9	1	*SYN1*	Synapsin I

List of the nine genes in cluster 1 with strong neuronal expression. A more complete list of genes in the three clusters is available in Table S1 in Additional data file 1.

Given that genes in cluster 1 were highly similar in their expression pattern, we asked whether they share potential *cis*-regulatory elements critical for neuronal-specific expression. Our strategy involved a systematic mapping of *cis*-regulatory elements for one gene (*Rab3A*), and then using these data as a guide to characterize the regulatory elements for other genes. *Rab3A *is well-suited for this case study for several reasons. First, the exclusive neuronal expression of *Rab3A *is conserved among mammalian species (mouse, rat and human), suggesting that common regulatory mechanisms may be evolutionarily conserved. Second, *Rab3A *is a small gene spanning a 3 kb genomic region with a 4.3 kb upstream intergenic region. This short genomic interval allows a systematic analysis of the entire region for functional regulatory elements. We followed a two-pronged approach to precisely characterize regulatory elements for the *Rab3A *gene: an assessment of multiple-species conserved elements (MCEs) and an unbiased screen for functional elements with a series of deletions.

### Identification of a genomic region sufficient for *Rab3A *neuronal-specific expression

**Figure 2:**
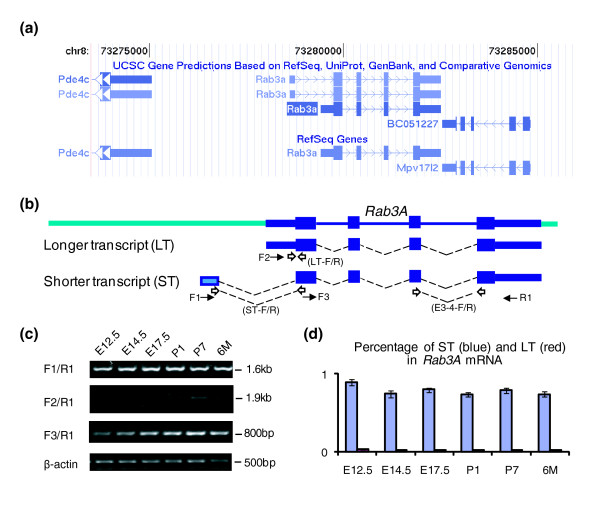
**Characterization of *Rab3A *transcripts**. **(a) **Illustration of the murine *Rab3A *locus in the University of California at Santa Cruz (UCSC) Genome Browser. Two *Rab3A *transcripts are shown in the top panel, among which the RefSeq transcript is represented as dark blue blocks. **(b) **Two splicing forms of *Rab3A *are labeled as LT (longer transcript) and ST (shorter transcript). The 5' UTR of *Rab3A *in the ST is shown as an unfilled box. The positions of primers used for RT-PCR are represented as solid arrows. Quantitative PCR primers are represented as open arrows and corresponding labels are in brackets. **(c) **Presence of *Rab3A *ST and LT examined by RT-PCR from mouse cortex tissues at various developmental stages. Primer set F1/R1 detects ST, primer set F2/R1 detects LT, and primer set F3/R1 reflects the total *Rab3A *level. *β-actin *level is used as control for RNA loading. Corresponding maker size is labeled on the right. **(d) **Percentage of ST and LT in total *Rab3A *mRNA was measured by quantitative RT-PCR from the same tissues used in (b). Specific primer sets were designed for ST and LT, respectively. A control primer set was used to detect total *Rab3A *level. All samples were tested simultaneously with the control set and the test set (either ST or LT). The ratio between ST and total RNA is presented as blue bars, and the ratio between LT and total RNA is presented as red bars. The error bars represent the standard deviation of the mean of four replicates used for each stage.

The *Rab3A *gene resides in one of the most gene-rich regions on mouse chromosome 8 between *Pd4c *and *BC051227 *(Figure 2a). Two *Rab3A *transcripts were found in the GeneBank mRNA database: a longer transcript containing a 374 bp 5' untranslated region (UTR) adjacent to the first exon, defined as the RefSeq transcript (build 37 assembly by NCBI); a shorter transcript containing a 156 bp 5' UTR 1,000 bp upstream of the first exon, reported in genome-wide transcriptome analysis [39] (Figure 2b). To validate these two transcripts, we examined the presence of the two distinct *Rab3A *mRNA in mouse brain by RT-PCR and quantitative PCR. Both transcripts were detected in mouse cortex at 12.5 days post coitum (dpc), 14.5 dpc, 17.5 dpc, postnatal day 1, postnatal day 7 and 6 months stages (Figure 2c). Quantitative analysis showed that the short transcript was the predominant form (Figure 2d). Although the RefSeq transcript corresponds to the long transcript, this mRNA was expressed at low level in all tested stages (Figure 2; Figure S1 in Additional data file 2).

**Figure 3:**
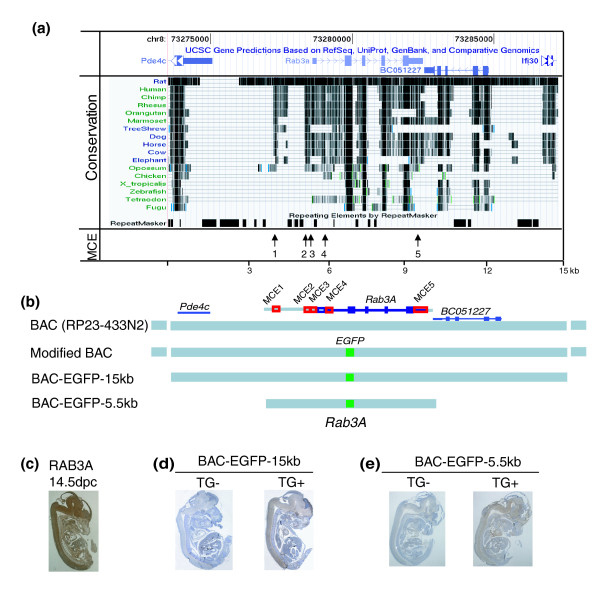
***In vivo *screen of genomic regions sufficient for *Rab3A *endogenous expression**. **(a) **Physical map of *Rab3A *locus on mouse chromosome 8, adapted from the UCSC Genome Browser. The top panel describes the 15 kb genomic region of the *Rab3A *locus. The middle track shows a measure of evolutionary conservation of the *Rab3A *locus in 17 vertebrates. Pairwise alignments of each species to the mouse genome are displayed as a grayscale density plot in which darker scale indicates higher conservation. The arrows on the bottom indicate the positions of multiple-species conserved elements (MCE1 to 5) around the *Rab3A *gene. **(b) **Schematic drawing of the EGFP reporter constructs used to generate transgenic mice. The two constructs (15 kb and 5.5 kb) were derived from the modified BAC clone RP23-433N2 containing an EGFP reporter (green block) in the position of *Rab3A *exon 1 (blue block). **(c) **Sagittal view of wild-type mouse embryos at 14.5 dpc stained with Rab3A antibody. A strong signal in the central nervous system (brain and spinal cord) was observed. **(d, e) **Sagittal view of transgenic embryos at 14.5 dpc stained with EGFP antibody. A strong signal in the central nervous system was observed in transgenic positive individuals, either carrying the 15 kb or 5.5 kb construct. TG-, transgenic negative; TG+, transgenic positive.

Based on this refined gene annotation, we initiated characterization of *Rab3A *regulatory elements by examining conserved elements in non-coding regions of the *Rab3A *locus. We compared the sequence of murine *Rab3A *to sequences of 17 vertebrate species using the UCSC genome browser [40]. The conservation profile revealed only five distinct MCEs in non-coding regions: MCE1 (-1,394, -1,216), MCE2 (-305, -137), MCE3 (-69, -2), MCE4 (+281, +500) and MCE5 (+3,381, +3,881), ranging from 76 to 82% nucleotide sequence identity between mammalian species (Figure 3a).

To examine if the 5.5 kb chromosome interval encompassing five MCEs around *Rab3A *was sufficient to give rise to neuronal expression of *Rab3A*, we tested this region by transient transgenesis using a modified bacterial artificial chromosome (BAC) clone. Specifically, the enhanced green fluorescent protein (EGFP) reporter gene replaced the first exon of *Rab3A *in a BAC clone (RP433N2) as described by [41]. The modified BAC clone was then truncated into two reporter constructs containing either a 15 kb genomic region (7 kb upstream and 5 kb downstream regions) or a 5.5 kb genomic region with all predicted MCEs (2.4 kb upstream and 100 bp downstream) (Figure 2b). Transgenic embryos were generated with each construct and assessed for EGFP expression at 14.5 dpc by histochemistry in three transgenic lines. Embryos carrying either the 5.5 kb or 15 kb genomic construct showed EGFP expression restricted to neural tissues, similar to that of endogenous *Rab3A *at the same stage (Figure 2c-e). The stronger signal in embryos carrying the 15 kb construct was likely due to high copy numbers of the EGFP reporter (data not shown). Based on the consistent neuronal expression of EGFP reporter, we concluded that the 5.5 kb region covering *Rab3A *coding sequence and five MCEs contained necessary *cis*-regulatory elements responsible for neuronal expression of *Rab3A*.

### Characterization of *cis*-regulatory elements in *Rab3A *locus by two strategies

**Figure 4:**
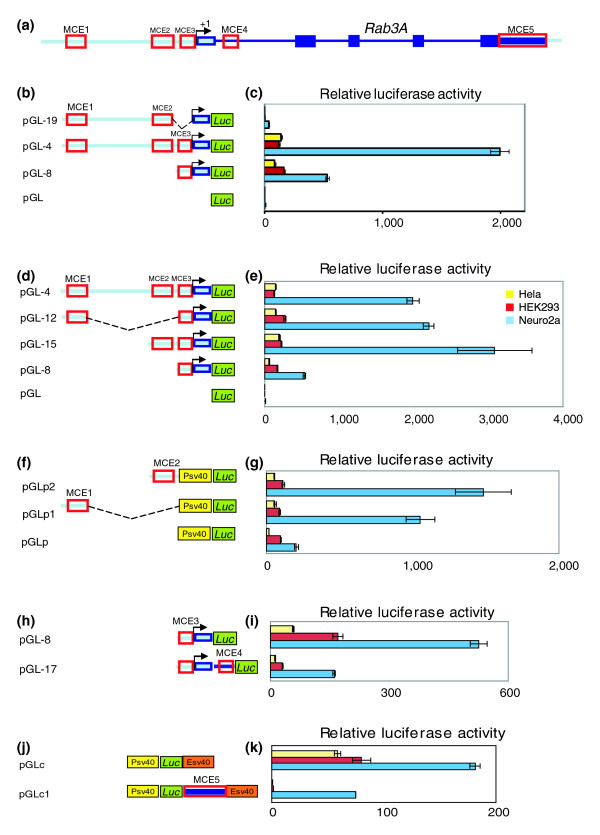
**Functional characterization of MCEs in the *Rab3A *locus by *Luciferase *assay**. **(a) **Gene structure of *Rab3A *is illustrated at the top and the transcription start site of the ST is designated as +1. **(b, c) **Presence of MCE3 induces *Luciferase *expression. The schematic representation of deletion constructs fused with pGL *Luciferase *reporter containing the *Firefly Luciferase *coding sequence (green block) but no promoter region. Corresponding *Luciferase *activity of each construct was measured in Neuro2a cells (blue bars), HEK293 cells (red bars) and Hela cells (yellow bars). The relative *Luciferase *activity was calculated by normalizing *Firefly Luciferase *activity to an internal control, *Renilla Luciferase *activity. Bars in the plot represent the mean of three independent experiments and the average standard deviation is indicated as error bars. **(d, e) **MCE1 and MCE2 increase *Luciferase *activity under the control of *Rab3A *promoter. **(f, g) **The effect of MCE1 and MCE2 is independent of the *Rab3A *promoter. The *Luciferase *construct of MCE1 or MCE2 was generated under the control of the SV40 promoter (yellow block). **(h, i) **MCE4 decreases *Luciferase *activity in three cell lines. (**j, k) **MCE5 under the control of the SV40 promoter (yellow block) and SV40 enhancer (orange block) decreases *Luciferase *activity in three cell lines.

*In vivo *transgenic experiments identified a 5.5 kb genomic region containing *cis*-regulatory elements responsible for neuronal expression of *Rab3A *at 14.5 dpc. To characterize regulatory elements, it was necessary to first define the *Rab3A *promoter region. *Luciferase *vector pGL, which contains the *Luciferase *coding sequence without a promoter, was used to identify the *Rab3A *promoter region. We generated a series of deletions in the 1.5 kb upstream region of *Rab3A *and fused them with pGL vector. These constructs were transfected into three cell lines: Neuro2a (mouse neuroblastoma), HEK 293 (human embryonic kidney) and Hela (human adenocarcinoma) to assess their ability to drive *Luciferase *expression (Figure 4b, c). The MCE3 region in pGL-8 initiated *Luciferase *expression in all three cell lines, whereas deletion of this region in pGL-19 completely disrupted *Luciferase *activity even with intact upstream enhancers. Therefore, the MCE3 region was defined as the *Rab3A *promoter region.

The remaining *Rab3A *MCEs were then examined for their ability to drive *Luciferase *expression under the control of the *Rab3A *promoter (MCE3) in three cell lines (Table 2). Two MCEs, MCE1 and MCE2, greatly enhance *Luciferase *activity in the Neuro2a cell line but not in HEK293 and Hela cells, supporting their roles as independent enhancers in a neuronal cell type specific manner (Figure 4d, e). A similar induction by MCE1 and MCE2 were also observed under the control of the SV40 promoter, ruling out the possibility that the *Rab3A *promoter itself contributed to the neuronal expression (Figure 4f, g). In contrast, the intronic MCE4 and the MCE5 in the 3' UTR led to decreased *Luciferase *activity in all tested cell lines, regardless of the promoter used (Figure 4h-k). Additional experiments will be needed to accurately define the role of these negative elements on *Rab3A *gene regulation; they may regulate transcription as repressors, or affect transcript stability, splicing or translational efficiency.

**Table 2:** Change of *Luciferase *activity due to multi-species conserved elements in different cell lines

Luciferase construct			Tested cell lines		
	
MCEs	Promoter	Enhancer	Neuro2a	HEK293	Hela
MCE1	MCE3		++	+/-	+
MCE1	SV40		++	+/-	+
MCE2	MCE3		+++	+/-	+
MCE2	SV40		+++	+/-	+
MCE4	MCE3		- -	- - -	- - -
MCE4	SV40	SV40	- -	- -	- -
MCE4	MCE3	Upstream position	+/-	+/-	NA
MCE5	MCE3	MCE1and MCE2	+/-	- -	- -
MCE5	SV40	SV40	- -	- - - -	- - - -

Summary of *Rab3A *multi-species conserved element (MCE) effects on *Luciferase *expression in Neuron2a, HEK293 and Hela cell lines. *Rab3A *MCE1, 2, 4 and 5 were examined individually by fusing into the *Luciferase *construct harboring either the *Rab3A *gene promoter (MCE3) or the SV40 promoter. Three cell lines were used: Neuro2a (mouse neuroblastoma), HEK 293 (human embryonic kidney) and Hela (human adenocarcinoma). MCE1 and MCE2 robustly enhanced *Luciferase *activity in the Neuro2a cell line but not at a comparable level in HEK293 and Hela cells under the control of either the MCE3 or SV40 promoter. MCE4 repressed *Luciferase *activity in all three cell lines, even under control of the SV40 enhancer. MCE4 also showed a strong positional effect, in which a position upstream of the MCE3 promoter failed to reduce *Luciferase *expression. MCE5 led to a decrease of *Luciferase *activity under the SV40 promoter and SV40 enhancer. However, when MCE1 and MCE2 induced *Luciferase *activity in Neuro2a cells, MCE5 did not have an effect on *Luciferase *activity. +, twofold increase; ++, fourfold increase; +++, sixfold increase; +/-, no change; - -, fourfold decrease; - - -, sixfold decrease; - - - -, approximately a tenfold decrease.

To complement the analysis of individual MCEs, we performed an unbiased screen of the 5.5 kb region for any potential *cis*-regulatory elements by *Luciferase *assay. Fine mapping of the entire 1.5 kb upstream region and the first intron was performed using a series of deletion constructs (Figure S2 in Additional data file 2). Closer inspection by additional *Luciferase *deletion constructs refined the core promoter region to a 64 bp region upstream of the *Rab3A *5' UTR (Figure S3 in Additional data file 2). Although two enhancer elements (Es) were mapped to E1 (-1,435, -1261) and E2 (-345, -123) regions, existence of repressor(s) (in the region between -802 and -346) cannot be excluded (Figure S4 in Additional data file 2). In addition, elements that reduce *Luciferase *activity (Ns) were found in the first intron, N1 (+240, +425) and N2 (+410, +556), and the *Rab3A *3' UTR (+3,362, +3,860) (Figures S5 and S6 in Additional data file 2). Strikingly, all experimentally identified regulatory elements correspond to MCEs. MCE1 (-1,394, -1,216) and MCE2 (-305, -137) closely correlated with the two enhancers, while MCE4 (+281, +500) covered the N1 region and overlapped with the N2 region. Thus, in the case of the *Rab3A *locus, MCEs in intergenic regions are good indicators of critical tissue-specific *cis*-regulatory elements.

Next we computationally searched for putative transcription factor binding sites (TFBSs) in the 2.8 kb genomic region (starting from1.5 kb upstream to the end of the first intron of *Rab3A*) using the PWM_SCAN tool [42] and the 546 positional weight matrices (PWMs) corresponding to vertebrate transcription factors in TRANSFAC version 8.4 [43]. Based on a *P*-value threshold of 0.0002, we predicted 77 and 23 binding sites in MCE1 and MCE2, respectively. These included well-known neuronal transcription factors NGF1-C, CREB, and EBF1/Olf-1 (Table S2 in Additional data file 1). In addition, MCE4 contained sites for transcription factors REST/NRSF (*P*-value = 0.0001), which might contribute to MCE4's repressor effect.

A group of binding sites located within a regulatory region may represent a CRM [44]. Therefore, we examined other genes that harbor the same CRM in their upstream regions for a similar expression pattern to that of the *Rab3A *gene. We searched 5 kb upstream regions of approximately 17,000 mouse RefSeq genes (version mm8) for the presence of the same set of binding sites as in *Rab3A *MCEs within a 500 bp window (see Materials and methods). This analysis identified 42 putative gene targets based on the *Rab3A *MCE1 CRM (Table S3 in Additional data file 1) and 13 gene targets based on the MCE2 CRM (data not shown). Next, we tested whether these 42 genes were expressed at significantly high levels in any of the mouse tissues for which genome-wide expression data are available. Based on the genome-wide gene expression profiles for 54 mouse tissues and 7 developmental stages (available in SymAtlas), we tested for each tissue, using the non-parametric Wilcoxon rank sum test (see Materials and methods), whether these 42 putative targets of the MCE1 CRM had a greater expression compared to all other genes. Overall, we found that these 42 genes had higher expression levels in 18 tissues with a *P*-value ≤ 0.05, of which 10 tissues were neural tissues such as cerebral cortex, preoptic area and substantia nigra. However, the 13 genes containing MCE2's CRM failed to show significant up-regulation in any of the mouse tissues tested. Our data suggest that at least a subset of CRMs, that is, for *Rab3A *MCE1, might be associated with a specific expression pattern.

### Identification of common motifs mediating neuronal-specific expression of the nine presynaptic genes

Next we hypothesized that co-expression of the nine presynaptic genes (*Rab3A*, *Rab3C*, *Scamp5*, *Snap25*, *Stxbp1*, *Syn1*, *SV2a*, *Camk2N1 *and DNM1) may be mediated via common CRMs. We have previously identified and characterized MCEs of 107 presynaptic genes [37]. Assuming that MCEs could serve as a reliable guide in the search for putative neuronal-specific CRMs, we searched for DNA-binding motifs enriched in the upstream MCEs near the nine genes with strong neuronal expression (cluster 1, Figure 1a) relative to the upstream MCEs near genes with nonspecific expression pattern (cluster 3, Figure 1c). This analysis revealed 16 PWMs enriched in cluster 1 genes' MCEs with a *P*-value ≤ 0.05 (Table 3); we also ensured that these predictions had low false discovery rates (FDRs; < 2.8%). These 16 motifs are among the TFBSs predicted in *Rab3A *MCE1 and MCE2, including binding sites for the well-known neuronal transcription factors REST/NRSF, CREB and NGF1c. Therefore, we asked if the presence of these sequence motifs is a likely determinant of neuronal-specific gene expression.

**Table 3:** Enriched transcription factor binding sites identified in cluster 1 multi-species conserved elements

Transcription factor	*P*-value	FDR (%)	Brief description
AP-2	0.002	0.08	Activating enhancer binding protein 2
ETF	0.006	0.23	TEA domain family member 2
CREB	0.008	0.32	cAMP responsive element binding protein
NRSF	0.008	0.33	RE1-silencing transcription factor
NGFI-C	0.009	0.36	Nerve growth factor/EGR4
MyoD	0.014	0.64	Myogenic differentiation 1
Olf-1	0.016	0.80	Olfactory neuronal TF 1
E2F	0.016	0.80	E2F family in control of cell cycle and tumor suppression
Myogenin	0.028	1.46	Myogenin/nuclear factor 1
NF-kappaB	0.028	1.46	Nuclear factor of kappa light polypeptide gene
c-Myc:Max	0.030	1.57	Myc proto-oncogene
Sp1	0.034	1.82	Trans-acting specific protein 1
AP-4	0.038	2.07	Activating enhancer binding protein 4
Nrf-1	0.039	2.08	Nuclear respiratory factor 1
HNF4	0.041	2.21	Hepatocyte nuclear factor 4
LXR	0.047	2.60	Liver × receptor

The enrichment of 16 transcription factor binding sites over-represented in MCEs from cluster 1 genes in comparison to those in cluster 2 or 3 genes with a *P*-value < 0.05. FDR, false discovery rate.

**Figure 5:**
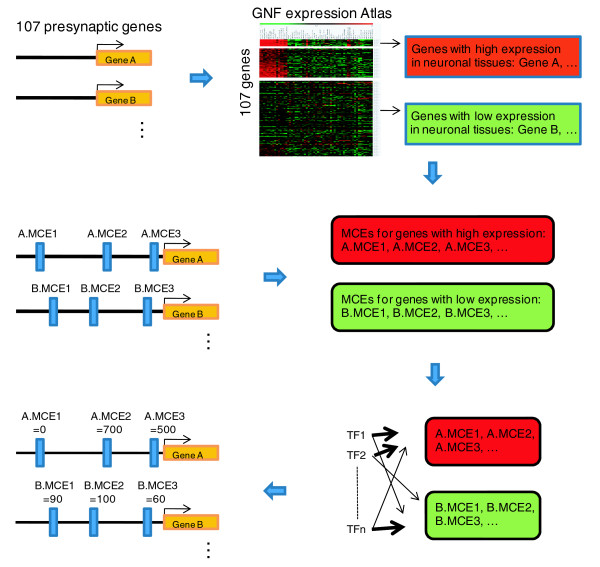
**General strategy to identify the common *cis*-regulatory modules for neuronal-specific expression and to score 3,347 MCEs from 107 presynaptic genes**. GNF, Genomics Institute of the Novartis Research Foundation.

Using the set of 16 transcription factor motifs over-represented in the cluster 1 genes' MCEs, we searched for 'neuronal-regulatory potential' in 3,347 intergenic MCEs corresponding to the 107 presynaptic genes (Figure 5). Specifically, we introduced a scoring function that scores each MCE as a sum of the likelihood-ratio of enrichment for each of the 16 motifs if they are present in the MCE. We ranked 3,347 MCEs by their scores representing the MCE's potential for regulating neuronal-specific expression in the range 0, for non-specific, to approximately 1,000, for the most neuronal-specific element. As expected, MCEs in cluster 1 have a greater score relative to those in cluster 2 (Wilcoxon rank sum *P*-value = 0.0015) and cluster 3 (Wilcoxon rank sum *P*-value = 2.2e-05) (Figure S7 in Additional data file 2). The differences remain significant even after we group all MCEs corresponding to a specific gene and use their median score as the summary statistic for the gene (both *P*-values ≤ 0.006). We note that while the above analysis is not meant to be an independent validation of our MCE ranking procedure, it does suggest that specific combinations of transcription factor motifs are enriched in MCEs in the vicinity of cluster 1 genes.

**Figure 6:**
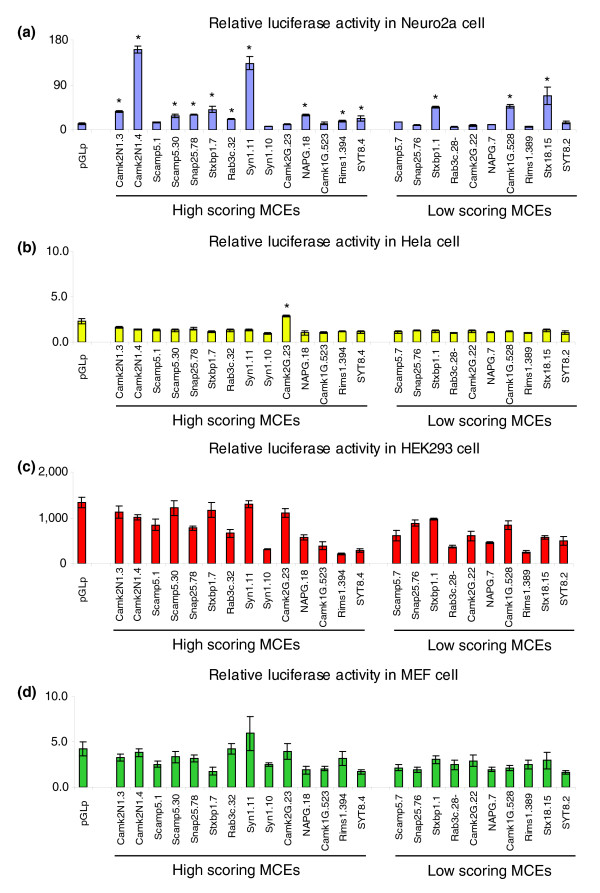
**Functional validation of 14 high scoring MCEs and 10 low scoring MCEs listed in Table 3**. *Luciferase *constructs were generated by inserting each MCE into the *Luciferase *promoter vector (pGL3p), which contains a SV40 promoter and *Luciferase *coding sequence. **(a-d) **The corresponding *Luciferase *activity was examined in one neuronal cell line, Neuro2a cells (a), and three non-neuronal cell lines, Hela cells (b), HEK293 cells (c) and MEF cells (d). Each construct was tested in triplicate in each set of experiments. Every experiment of all 24 constructs was repeated at least three times. Each *Luciferase *construct was co-transfected with the *Renilla *pRLCMV vector for normalization. MCEs that significantly induced *Luciferase *activity are highlighted with an asterisk (*P *< 0.05, Student's *t*-test). The error bars represent the standard deviation of the mean of triplicates used for each construct.

Using this scoring strategy, we selected candidates of neuronal-specific enhancers from a pool of 3,347 intergenic MCEs for experimental validation. Apart from already tested *Rab3A *elements (MCE1 and MCE2), we selected 14 high-scoring MCEs (ranging from 1,006 to 90.5) for 12 genes (Table 4; see Materials and methods). Of the fourteen MCEs, eleven belong to cluster 1, two belong to cluster 2, and three belong to cluster 3 genes. In addition, as a negative control, for ten selected genes a low scoring MCE (score = 0) was also randomly chosen, matched with the high score MCE with regard to length and distance to target gene. We assessed the regulatory role of 24 MCEs (14 high scoring and 10 low scoring MCEs) in the *Luciferase *promoter assays, in which the *Luciferase *vector contained a SV40 promoter fused with the coding sequence of the firefly *Luciferase *gene. Tested cell lines included Neuro2a (mouse neuroblastoma cell), MEF (mouse embryonic fibroblast cell), HEK293 (human embryonic kidney cell 293) and Hela (human adenocarcinoma cell). Overall, including the two *Rab3A *MCEs (MCE1 and MCE2), we observed robust enhancer activities driven by 75% (12 of 16) of the high-scoring MCEs and in 30% (3 of 10) of the low-scoring MCEs in Neuro2a cells; none of the tested MCEs increased *Luciferase *expression in MEF, HEK293 and Hela cells except CAMK2G.23, which slightly induced *Luciferase *expression in Hela cells (Figure 6). Therefore, a majority (12 MCEs; 75%) of predicted neuronal enhancers revealed a regulatory role in the neuronal cell type. More specifically, of the total 26 MCEs tested, 12 were true positives, 4 were false positives, 7 were true negatives and 3 were false negatives (Table 4). This yields a nominal sensitivity of 12/15 = 80% and a nominal specificity of 7/11 = 63%. Our results, although based on a small testing set, indicate that this scoring strategy based on expression similarity and motif-enrichment provides a powerful tool for the identification of enhancers in a particular tissue of interest, in our case, neuronal tissue/cell type.

**Table 4:** High scoring and corresponding low scoring multi-species conserved elements

Cluster	Gene	MCE	Score	Control	Score
1	*CAMK2N1*	**CAMK2N1.3**	548.29	No control MCE*	
		**CAMK2N1.4**	152.32		
1	*SCAMP5*	SCAMP5.1	476.64	SCAMP5.7	0
		**SCAMP5.30**	90.5		
1	*SNAP25*	**SNAP25.78**	479.98	SNAP25.76	0
1	*STXBP1*	**STXBP1.7**	479.61	**STXBP1.1**	0
1	*RAB3C*	**RAB3C.32**	96.13	RAB3C.28-9	0
1	*SYN1*	**SYN1.11**	1006.13	No control MCE*	
		SYN1.10	234.92		
1	*RAB3A*	**RAB3A.MCE1**	997.88	No control MCE*	
		**RAB3A.MCE2**	98.63		
2	*CAMK2G*	CAMK2G.23	520.43	CAMK2G.22	0
2	*NAPG*	**NAPG.18**	522.09	NAPG.7	0
3	*CAMK1G*	CAMK1G.523	639.59	**CAMK1G.528**	0
3	*RIMS1*	**RIMS1.394**	680.61	RIMS1.389	0
3	*STX18*	STX18.12*	627.98	**STX18.15**	0
3	*SYT8*	**SYT8.4**	508.56	SYT8.2	0

The list of 16 high scoring multi-species conserved elements (MCEs) and 10 low scoring MCEs for experimental validation (see Materials and method for selection detail). *Genomic regions upstream of *CAMK2N1*, *SYN1 *and *Rab3A *genes do not contain low-scoring MCEs. One of the high-scoring MCEs (STX18.12) failed to be cloned. A *Luciferase *assay in the Neuro2a cell line identified neuronal enhancers in 12 high-scoring MCEs and 3 low-scoring MCEs marked in bold (Student's *t*-test, *P *< 0.05).

## Discussion

Deciphering the *cis*-regulatory code for tissue-specific and developmental-stage-specific gene expression remains a significant challenge [44-48]. In this study we have focused on identifying common CRMs mediating neuronal expression, using a combined computational and experimental approach. In order to achieve biological specificity, we have restricted our investigation to neuronal-specific genes of a specific function, namely, presynaptic neurotransmitter release. Using a combination of TFBSs in conserved elements in nine genes with an abundant and restricted expression in neuronal tissues, we developed a combined computational-experimental strategy for the evaluation of the 'neuronal regulatory potential' of MCEs.

Several slightly different approaches to identify CRMs mediating tissue-specific gene expression have been previously proposed [44-48]. Our approach differs from these in a few important ways. For instance, some of the other approaches employed genome-wide selection of co-expressed genes solely based on microarray expression profiles. This may recruit many genes that may belong to diverse pathways, therefore reducing the signal-to-noise ratio in subsequent analysis [49]. In contrast, we restricted our analysis to 150 genes known to be involved in synaptic transmission in presynaptic neurons; we further divided them into distinct expression clusters based on neuronal-related and non-neuronal-related tissue types. Nine presynaptic genes were found to have a striking neuronal-specific pattern that is likely co-regulated by common CRMs, whereas 72 genes with widespread and/or low levels of expression served as an internal control.

A major challenge in the identification of TFBSs is that the binding motifs are usually short and degenerate and thus result in high false positive rates [32]. However, it has been shown that functional binding sites tend to be clustered [45]. Thus, by first identifying enriched motifs in the MCEs near the nine neuronal-specific genes, and by focusing on clustered occurrences of these motifs, we could reduce the rate of false positives. Seventy-two presynaptic genes with non-specific expression served as negative controls, further enhancing the specificity of CRM identification.

Another challenging issue with many previous computational studies is the arbitrary selection of the proximal promoter region to search for enriched motifs and determine CRMs [44]. As previously shown, selection of evolutionarily conserved sequences from larger intergenic or intronic (MCE) regions may represent an alternative approach [44]. Consistent with these previous findings, a detailed experimental delineation of regulatory elements of *Rab3A *revealed a remarkable correspondence between functional elements and evolutionary conservation. However, it has been shown that, in many cases, functional elements are known to reside in non-conserved regions [50,51].

To capture the contributions of various enriched motifs comprising a CRM, we chose a likelihood-ratio-based scoring metric specifically designed to assess the neuronal regulatory potential of a MCE. Our scoring metric weights the presence of a binding site based on its enrichment *P*-value, such that more enriched (lower *P*-value) motifs are weighted higher. This choice was based on the ability of the scoring metric to discriminate MCEs in the vicinity of genes expressed highly and specifically in neuronal cell types from MCEs near genes that are broadly expressed. In this respect, our approach is most similar to that taken in [44], where the authors estimated the weights based on an optimization procedure. Besides the statistical analysis used to verify the specificity of the MCE ranking procedure, we experimentally validated several high-scoring and low-scoring MCEs through a cell line-based *Luciferase *reporter assay. The comparison of neuronal cell line and non-neuronal cells offered, to some extent, a clue as to tissue/cell type specificity. Overall, based on 26 tested MCEs, we were able to predict neuronal-specific expression with roughly 75% sensitivity and 63% specificity. Although we have provided a proof-of-principle, we expect that a scoring metric trained on a larger set of validated neuronal-specific genes would permit a genome-wide prediction of neuronal enhancers.

From a methodological perspective, the strengths of our study include: carefully selected co-expressed genes with a related function (pathway); a two-pronged approach to determine *cis*-regulatory elements of one prominent gene - *Rab3A*; reliance on MCEs regardless of their proximity to the gene; pre-determination of enriched motifs to define the CRM; a weighted scoring strategy to rank MCEs according to their neuronal regulatory potential; and experimental validation of a subset of both high-scoring and low-scoring MCEs for their neuronal-specific enhancer properties. Certainly, despite these advantages, we are also aware of several limitations. First, our experimental validation is based on cell-culture experiments. However, *in vitro *methods cannot fully recapitulate endogenous expression patterns. As described in Pennacchio *et al*. [27], an *in vivo *enhancer assay in transgenic mouse embryos would be a more reliable and conclusive method to evaluate our findings. Moreover, although our scoring system is applicable to a genome-wide prediction of neuronal-specific enhancers, we still need to assess this application on a large set of gene and genomic elements.

This study focuses on identification of *cis*-regulatory elements responsible for neuronal expression; however, *cis *elements only partly determine gene expression, which is regulated by additional epigenetic factors such as nucleosome positioning, DNA methylation and a number of histone modifications [52-54]. Such epigenetic marks play critical roles in gene regulation in higher organisms. For instance, several studies have revealed the critical role of nucleosomes in chromatin structure and remodeling and, ultimately, in gene regulation. Nucleosome occupancy can block access to regulatory elements, thereby inhibiting the binding of transcription factors to specific DNA sequences. To assess the role of nucleosome occupancy, using a previously published computational modeling approach and the software program provided by the authors [55], we predicted the nucleosome occupancy probability for the 16 MCEs (including 50 bp flanking sequences). In our study, four MCEs with high scores for enrichment of neuronal-specific motifs did not show any enhancement of *Luciferase *gene expression. We found that relative to the 12 positive elements, the 4 negative elements had significantly greater probability of being occupied by nucleosomes (Mann-Whitney rank sum *P*-value = 0.04). This finding suggests that nucleosome occupancy, in addition to other levels of regulation, needs to be included in the evaluation of regulatory potential of genomic elements.

Conserved non-coding sequences are not only essential for gene expression, but are also associated with phenotypic variability and human disorders. To date, increasing attention has been focused on changes in *cis*-regulatory regions, such as substitutions or deletions, that might contribute to species uniqueness and human disorders [24,56,57]. Several *cis*-regulatory mutations are known to underlie diverse aspects of behavior, physiology and disease susceptibility in human [58-60]. For example, a non-coding single nucleotide polymorphism (RET+3) within a conserved enhancer element in the first intron of RET, a receptor tyrosin kinase, has been reported to be significantly associated with Hirschsprung disease featured by congenital aganglionosis with megacolon [61-63]. Our study decoding *cis*-regulatory elements required for neuronal gene expression could further facilitate investigation of genetic variations in functional regulatory elements, thus greatly improving our knowledge of how regulatory sequences are involved in human diseases.

## Conclusions

We selected nine presynaptic genes that were most abundantly expressed in neural tissues and demonstrated, by *in vivo *and *in vitro *screens, that MCEs upstream of one of these genes, *Rab3A*, functioned as *cis*-regulatory elements. We then identified 16 transcription factor binding motifs that were enriched in intergenic MCEs in the vicinity of these nine genes. We devised a computational scoring metric based on the enriched motifs to assess an MCE's potential to function as a neuronal-specific enhancer. This scoring metric was shown to accurately detect neuronal-specific enhancers, based on experimental validation of a number of predicted MCEs using cell based assays. Thus, our study introduces a comprehensive strategy for identification of neuronal specific enhancers.

## Materials and methods

### Expression clustering of microarray profiles

Mouse expression profiles are available from the Genomics Institute of the Novartis Research Foundation [38]; the data used were based on the analysis across 54 mouse tissues and 7 developmental stages on Affymetrix microarrays [64]. Normalized and filtered expression files were analyzed using TIGR Multiexperiment Viewer (MeV), a versatile microarray data analysis tool that incorporates algorithms for clustering, visualization, and statistical analysis [65,66]. We clustered 240 unique oligonucleotide probe sets that interrogated 126 different presynaptic genes into three distinct clusters. By closer inspection, we excluded 52 probes that hybridized to intergenic or intronic sequences. In addition, clustering of expression data placed 19 genes into more than one cluster and these genes were eliminated in the further analysis. As a result, 107 genes corresponding to 161 probes were clustered into 3 distinct expression groups by the hierarchical k-means clustering method.

### RT-PCR and quantitative PCR

Brain tissues or cultured cells were homogenized in Trizol (Invitrogen, Carlsbad, CA, USA), and total RNA was extracted by the Trizol procedure and using an RNeasy mini prep kit (Qiagen, Valencia, CA, USA). For RT-PCR, 2 μg aliquots of DNase-treated RNA were reverse-transcribed using a High Capacity cDNA Archive kit (Applied Biosystems, Foster City, CA, USA) as described by the manufacturer.

Reverse-transcribed products (2 to 4 ng) were used for PCR and the products obtained after 26 or 32 PCR cycles with the different primers were analyzed by agarose gel. Primer set F1/R1 was designed to be specific for the shorter transcript of *Rab3A *(ST), F2/R1 was designed to be specific for the longer transcript of *Rab3A *(LT), and F3/R1 was targeted to common sequences of both the ST and LT as total *Rab3A *mRNA. The β-actin set was the control for RNA loading (Table S4 in Additional data file 1).

cDNA products (2 to 4 ng) were used for quantitative PCR with a SYBR green PCR kit (Applied Biosystems). The primer set ST-F/R was used to detect the ST, the primer set LT-F/R to detect the LT, and the primer set E3-4-F/R to detect exons 3 and 4 of the total *Rab3A *mRNA. All samples were tested simultaneously with two primer sets: the control primer set (E3-4-F/R) and the test primer set (either ST-F/R or LT-F/R). This allowed ST/LT expression levels to be normalized to the total *Rab3A *level. All samples were tested in triplicate. Relative quantification (quantitative PCR) was performed on an ABI Prism 7900HT system and Ct values were analyzed by SDS2.2 software (Applied Biosystems). The relative mRNA quantity of ST and LT at each developmental stage was normalized to total *Rab3A *mRNA to obtain the relative ratio according to the calculation method in the user manual.

### Generation of EGFP reporter constructs

The EGFP reporter gene was introduced into BAC clone RPCI-23 433N2 (CHORI, BAC/PAC Resources, Oakland, CA, USA) by homologous recombination in *Escherichia coli *according to the method of Gong *et al*. [41]. Two 500 bp sequences (homology arms A and B) flanking mouse *Rab3A *exon 1 were amplified by PCR (Table S4 in Additional data file 1) and cloned into the *Asc*I and *Pac*I restriction sites flanking the EGFP coding sequence in the pLD53SCAEB shuttle vector. The modified vector was transformed into BAC host cell DH10B by electroporation. After two-step homologous recombination, the modified BACs were screened by PCR (Table S4 in Additional data file 1) to detect the two EGFP junctions and confirmed by Southern blot. Specifically, DNA was digested with *Eco*RI or *Hin*dIII, separated by electrophoresis on a 0.8% agarose gel and transferred to a nylon membrane. The blots were analyzed using the 'A box' or 'EGFP' as probe. Wild-type BAC DNA served as the negative control and shuttle vector as the positive control.

The BAC-EGFP 15 kb construct was generated from the modified BAC clone. The modified BAC was digested with *Not*I and *Swa*I (Roche Applied Science, Indianapolis, IN, USA) and separated by electrophoresis on a 0.8% agarose gel. The 15 kb fragments were enriched by gel extraction and cloned into the *Not*I site of the pBSKS^+ ^vector. Resultant colonies were screened by the presence of EGFP gene (Table S4 in Additional data file 1) and positive clones were confirmed by DNA sequencing. The BAC-EGFP 5.5 kb construct was directly amplified by PCR (Table S4 in Additional data file 1) upon the modified BAC and then cloned into the *Eco*RV site of the pBSKS+ vector. Resultant colonies were screened by the presence of EGFP and positive clones were confirmed by DNA sequencing. The two reporter constructs were linearized by *Not*I and *Sal*I and used to generate transgenic mouse lines.

### Generation and genotyping of transgenic mice

By pronuclear microinjection, reporter constructs were inserted into fertilized eggs derived from the intercross of the (BL6xSJL) F1 mouse strain (Transgenic Core Facility, University of Pennsylvania). Transgenic embryos were collected at 14.5 dpc. Tail snips of transgenic embryos were incubated overnight in 700 μl of lysis buffer (10 mM Tris, pH 8; 10 mM EDTA, pH 8; 0.1 M NaCl; 2% SDS) supplemented with 1 μg/μl proteinase K (Sigma-Aldrich, St. Louis, MO, USA). DNA was extracted using standard phenol-chloroform procedures, precipitated with ethanol, and dissolved in 10 mM Tris/10 mM EDTA. PCR was performed to determine the presence of the EGFP gene.

### Immunohistochemistry of transgenic positive embryos

Transgenic embryos (three transgenic positives and three negatives) at 14.5 dpc were fixed in 4% paraformaldehyde overnight at 4°C, dehydrated using graded alcohols and embedded in paraffin. Five-micron sections were deposited onto superfrost-coated slides and air dried. After heating at 65°C for 20 minutes, slides were deparaffinized in three rounds of xylene. Endogenous peroxidase activity was blocked with incubation in 100% methanol with 1% H_2_O_2 _for 20 minutes at room temperature. Slides were rehydrated using graded alcohol at room temperature and placed into a humidifier. After slides were bathed with blocking buffer (10% horse serum, 0.1% tween-20, dilute with 1× phosphate-buffered saline) for 30 minutes at room temperature, each slide was then covered with the primary anti-GFP antibody (15 μg/ml; #11122, Invitrogen) and left overnight at 4°C. A Biotin-Streptavidin Amplified kit (Biogenex Laboratories Inc, San Ramon, CA, USA) was then used as follows. Incubation of biotinylate secondary anti-rabbit antibody was followed by application of streptavidin conjugated horse radish peroxidase labeled antibody. Each incubation lasted 30 minutes at room temperature and phosphate-buffered saline was used as wash. The DAB chromogen was applied for 5 minutes (color reaction product: brown). The slides were then counterstained with hematoxylin, dehydrated and covered by a coverslip. All images were visualized using conventional microscopy.

### Generation of *Luciferase *constructs

*Rab3A*-related *Luciferase *constructs were generated by inserting the genomic sequence of the *Rab3A *locus into the *Sma*I site of the *Firefly Luciferase *pGL3-basic vector (pGL series), pGL3-promoter vector (pGLp series) or pGL3-control vector (pGLc series) (Promega Corporation, Madison, WI, USA). PCR was used to generate insertion fragments using related primer sets listed in Table S4 in Additional data file 1. MCE-related *Luciferase *constructs were generated by inserting each MCE into the *Sma*I site of the *Firefly Luciferase *pGL3-promoter vector. MCEs were amplified by PCR using related primer sets listed in Table S4 in Additional data file 1. All *Luciferase *constructs were confirmed by sequencing.

### Transient transfection and *Luciferase *assays

Cells were plated in 6-well plates and grown to 60% confluence in growth medium (Dulbecco's modified Eagle's medium (DMEM), 5% fetal bovine serum, 1% L-glutamin, 1% MEM Non-essential Amino Acid solution, 1% antibiotics; Sigma-Aldrich, St. Louis, MO, USA). Cells were co-transfected with *Firefly Luciferase *constructs and *Renilla Luciferase *pRLCMV [pRLCMVrenilla] vector (Promega Corportaion) using the liposome-mediated Fugene 6 Reagent (Roche Applied Science,) at a DNA/lipid ratio of 2:1 in DMEM medium without fetal bovine serum. On the day of the transfection, 3 μg of *Firefly Luciferase *construct DNA and 0.06 μg of *Renilla Luciferase *vector were mixed with 6 μl of Transfast reagent and incubated at room temperature for 20 minutes. After incubation, plated cells were changed with fresh modified growth medium (DMEM, 1% fetal bovine serum, 1% L-glutamin, 1% MEM Non-essential Amino Acid solution, 1% antibiotics; Sigma-Aldrich) and overlaid with DNA/Transfast mixture. Cells were incubated for 48 hours and harvested with 500 μl of passive lysis buffer (Promega Corporation). Luciferase activities were measured with 20 μl of protein extract solution using the dual-luciferase reporter assay system (Promega Corporation) and a Bio-Rad luminometer (Bio-Rad, Hercules, CA, USA). Each construct was tested in triplicate in each set of experiments. The ratio between the *Firefly Luciferase *and *Renilla Luciferase *was used as the relative *Luciferase *activity for each construct. Every experiment for all 24 constructs was repeated at least three times. For experimental validation of MCEs, we used Student's *t*-test to calculate statistical significance, in which a *P*-value < 0.05 was considered to be significant.

### Identification of target genes sharing the CRMs of the *Rab3A *MCEs and their tissue-specific expression

Based on putative TFBSs in *Rab3A *MCE1, we first merged all overlapping binding sites whose corresponding PWMs were 'similar'. For PWM-pair similarity we used a previously published metric based on relative entropy and applied a cutoff *P*-value of 0.02 [67]. Among all merged hits we retained the best scoring binding site. This yielded seven PWMs for MCE1. We then expanded each of the seven PWMs to include other related PWMs, using the same operational definition of similarity as above; we thus had seven families of PWMs. Based on genome-wide annotation of putative binding sites using our previously described phylogenetic footprinting approach [42], we searched for additional mouse transcripts that harbored at least one member of each of the seven PWM families within a 500 bp window in their 5 kb upstream region. We thus identified 42 transcripts. Using the same strategy, MCE2 also yielded 7 families of related PWMs and 13 gene targets were identified by this CRM in the genome-wide search.

We downloaded the genome-wide expression profiles for 61 tissues from Novartis. We specifically obtained the GCRMA (Guanine Cytosine Robust Multi-Array Analysis) processed expression values. For each tissue, using the Wilcoxon rank sum test, we tested the null hypothesis that the expression levels of these gene targets were no greater than those of the other genes in that tissue.

### Identification of over-represented motifs in cluster 1 genes

The MCEs of presynaptic genes were defined by Hadley *et al*. [37]. Using the computational tool phastCons [68], MCEs were defined as the most conserved elements from genome-wide alignments of the mouse genome with seven other vertebrate genomes, including human, chimpanzee, dog, rat, chicken, zebra fish and puffer fish.

Using our PWM_SCAN tool and TRANSFAC PWMs as described above, we identified the putative binding sites in all MCEs upstream of the 107 presynaptic genes until the next neighboring gene. For each motif, we compared the number of occurrences in the MCEs corresponding to cluster 1 genes relative to the MCEs corresponding to cluster 3 genes and estimated the significance of enrichment using 1,000 random permutations.

We then estimated the FDR for each *P*-value threshold based on permutations. From the entire set of MCEs that was used as a background control for the above enrichment analysis, we randomly selected 158 MCEs (same number as the cluster 1 5' MCEs used for enrichment analysis) and estimated the enrichment of 546 PWMs. Based on 100 randomizations, we have, in effect, done 54,600 tests of enrichment. Since *a priori *we do not expect any meaningful enrichment in these randomly selected MCEs, the fraction of tests that qualify a certain *P*-value threshold provide an estimate of the FDR for that *P*-value.

### *Cis*-regulatory module scoring metric

Given the 16 motifs enriched in cluster 1 MCEs, we hypothesized that the co-occurrence of some of these motifs in an MCE may be indicative of the MCE's role as a neuronal-specific enhancer. However, we wished to weight motifs proportional to their enrichment score. For a motif with a Fisher exact test *P*-value of *p*, we set its weight as (1 - *p*)/*p*, which can be interpreted as the likelihood ratio of the probability of the motif not occurring by chance to the probability of the motif occurring by chance. Let *p*(*x*) be the *P*-value of motif *x*. Thus, for an MCE having occurrences of motifs *m1*, *m2*, ..., *mk*, the score is given by:



### Selection of 16 MCEs for *Luciferase *assay

Approximately 3,347 upstream MCEs from 107 presynaptic genes were ranked by scores. We selected the top 30 MCEs, corresponding to 12 presynaptic genes. Among the 12 genes, 6 belong to cluster 1, 2 belong to cluster 2 and 4 belonged to cluster 3. For each gene, normally one MCE with the highest score was chose for evaluation. Two MCEs (one with the highest score and one with a lower score) were chosen only if the gene was a cluster 1 gene and had several MCEs in the top 30. To complete the cluster 1 gene list, a low score MCE (score = 96.13) of the *Rab3C *gene was also chosen. All selected MCEs were carefully inspected with regard to their conservation, relative position to neighboring genes and length. In addition, as a negative control, for each selected gene a zero scoring MCE was randomly chosen, matched to the high score MCEs with regard to their length and distance to the target gene. (Exceptions were *CAMK2N1*, *SYN1 *and *Rab3A*, which did not have a zero scoring MCE, and one of the 17 high scoring MCEs (STX18.12) failed to be cloned.) Experimental validation using the *Luciferase *assay was as described above.

## Abbreviations

BAC: bacterial artificial chromosome; CRM: *cis*-regulatory module; dpc: days post coitum; DMEM: Dulbecco's modified Eagle's medium; E: enhancer element of *Rab3A *gene; EGFP: enhanced green fluorescent protein; FDR: false discovery rate; GCRMA: Guanine Cytosine Robust Multi-Array Analysis; LT: longer transcript of *Rab3A*; MCE: multi-species conserved element; N: element with a negative function in *Rab3A *gene expression; PWM: positional weight matrix; ST: shorter transcript of *Rab3A*; TFBS: transcription factor binding site; UTR: untranslated region.

## Authors' contributions

RL carried out the clustering analysis of presynaptic genes, performed experiments and drafted the manuscript. SH conducted CRM analysis and designed the scoring strategy. MB conceived of the study, and participated in its design and coordination. All authors contributed to the writing of the paper, and read and approved the final manuscript.

## Additional data files

The following additional data are available with the online version of this paper: Tables S1 to S4 (Additional data file 1); Figures S1 to S7 (Additional data file 2).

## Supplementary Material

Additional data file 1 Table S1 lists 107 presynaptic genes in corresponding expression clusters. Table S2 lists TFBSs identified in *Rab3A *MCEs. Table S3 lists 42 additional genes potentially regulated by *Rab3A *ECR1's CRM and their expression levels. Table S4 lists all primer sets used in this study. Click here for file

Additional data file 2 Figure S1 shows *Rab3A *amplification in cDNA and genomic DNA (gDNA) samples. Figure S2 shows the screen of functional elements in the non-coding region of the *Rab3A *locus. Figure S3 shows fine mapping of the promoter region of *Rab3A*. Figure S4 shows the screen of the 1.5 kb upstream region of *Rab3A *for potential regulatory element(s). Figure S5 shows the screen and characterization of the first intron of *Rab3A *for regulatory element(s). Figure S6 shows the masking of MCE5 by the presence of MCE1 and MCE2 in Neuro2a cells. Figure S7 shows the statistical evaluation of the scoring strategy for MCEs. Click here for file
