# Supplementary material for: Motifs and cis-regulatory modules mediating the expression of genes co-expressed in presynaptic neurons
Source: Genome Biol. 2009 Jul 1;10(7):R72. doi: 10.1186/gb-2009-10-7-r72 (PMC2728526; doi:10.1186/gb-2009-10-7-r72)
Supplement: Additional data file 1 — Table S1 lists 107 presynaptic genes in corresponding expression clusters. Table S2 lists TFBSs identified in Rab3A MCEs. Table S3 lists 42 additional genes potentially regulated by Rab3A ECR1's CRM and their expression levels. Table S4 lists all primer sets used in this study. [file gb-2009-10-7-r72-S1.doc]

**Additional data file 1**

Table s1. List of 107 presynaptic genes in corresponding expression clusters.

| **Clustering of 107 murine presynaptic genes** | | | | | |  |  |  |  |  |
| --- | --- | --- | --- | --- | --- | --- | --- | --- | --- | --- |
| Cluster | Gene | # |  | Cluster | Gene | # |  | Cluster | Gene | # |
| 1 | *CAMK2N1* | 1 |  | 3 | *APBA1* | 1 |  | 3 | *SLC30A3* | 37 |
| 1 | *DNM1* | 2 |  | 3 | *ASPM* | 2 |  | 3 | *SLC30A4* | 38 |
| 1 | *RAB3A* | 3 |  | 3 | *BSN* | 3 |  | 3 | *SNAPAP* | 39 |
| 1 | *RAB3C* | 4 |  | 3 | *CALM1* | 4 |  | 3 | *STX11* | 40 |
| 1 | *SCAMP5* | 5 |  | 3 | *CALML3* | 5 |  | 3 | *STX16* | 41 |
| 1 | *SNAP25* | 6 |  | 3 | *CALML4* | 6 |  | 3 | *STX17* | 42 |
| 1 | *STXBP1* | 7 |  | 3 | *CAMK1G* | 7 |  | 3 | *STX18* | 43 |
| 1 | *SV2A* | 8 |  | 3 | *CAMK2D* | 8 |  | 3 | *STX4A* | 44 |
| 1 | *SYN1* | 9 |  | 3 | *CAMK4* | 9 |  | 3 | *STX5A* | 45 |
| 2 | *APBA2* | 1 |  | 3 | *CASK* | 10 |  | 3 | *STX6* | 46 |
| 2 | *BZRAP1* | 2 |  | 3 | *DMXL2* | 11 |  | 3 | *STX7* | 47 |
| 2 | *CALM2* | 3 |  | 3 | *EPIM* | 12 |  | 3 | *STX8* | 48 |
| 2 | *CALM3* | 4 |  | 3 | *EXOC1* | 13 |  | 3 | *STXBP2* | 49 |
| 2 | *CAMK1* | 5 |  | 3 | *EXOC2* | 14 |  | 3 | *STXBP4* | 50 |
| 2 | *CAMK2G* | 6 |  | 3 | *EXOC4* | 15 |  | 3 | *SYN3* | 51 |
| 2 | *CAMK2N2* | 7 |  | 3 | *EXOC6* | 16 |  | 3 | *SYNGR2* | 52 |
| 2 | *EXOC3* | 8 |  | 3 | *EXOC7* | 17 |  | 3 | *SYNGR4* | 53 |
| 2 | *GDI1* | 9 |  | 3 | *EXOC8* | 18 |  | 3 | *SYP* | 54 |
| 2 | *NAPG* | 10 |  | 3 | *GZMB* | 19 |  | 3 | *SYT10* | 55 |
| 2 | *NLGN2* | 11 |  | 3 | *NAPA* | 20 |  | 3 | *SYT12* | 56 |
| 2 | *NLGN3* | 12 |  | 3 | *RAB3D* | 21 |  | 3 | *SYT14* | 57 |
| 2 | *PCLO* | 13 |  | 3 | *RAB3GAP1* | 22 |  | 3 | *SYT15* | 58 |
| 2 | *RAB3B* | 14 |  | 3 | *RAB5A* | 23 |  | 3 | *SYT2* | 59 |
| 2 | *RPH3A* | 15 |  | 3 | *RAB5B* | 24 |  | 3 | *SYT3* | 60 |
| 2 | *STX1A* | 16 |  | 3 | *RAB5C* | 25 |  | 3 | *SYT6* | 61 |
| 2 | *STX1B2* | 17 |  | 3 | *RAB6IP2* | 26 |  | 3 | *SYT7* | 62 |
| 2 | *SVOP* | 18 |  | 3 | *RABAC1* | 27 |  | 3 | *SYT8* | 63 |
| 2 | *SYNGR3* | 19 |  | 3 | *RABGEF1* | 28 |  | 3 | *SYTL1* | 64 |
| 2 | *SYT16* | 20 |  | 3 | *RABGGTA* | 29 |  | 3 | *SYTL2* | 65 |
| 2 | *SYT5* | 21 |  | 3 | *RABGGTB* | 30 |  | 3 | *SYTL4* | 66 |
| 2 | *SYT9* | 22 |  | 3 | *RABIF* | 31 |  | 3 | *UNC13B* | 67 |
| 2 | *UNC13C* | 23 |  | 3 | *RIMS1* | 32 |  | 3 | *UNC13D* | 68 |
| 2 | *VAMP1* | 24 |  | 3 | *RIMS3* | 33 |  | 3 | *VAMP3* | 69 |
| 2 | *VAMP2* | 25 |  | 3 | *SCAMP2* | 34 |  | 3 | *VAMP5* | 70 |
| 2 | *VAMP4* | 26 |  | 3 | *SCAMP3* | 35 |  | 3 | *VAMP8* | 71 |
| 2 | *SYT13* | 27 |  | 3 | *SCAMP4* | 36 |  |  |  |  |

Table s2. Transcription factor binding sites identified in *Rab3A* MCEs. PWM_SCAN tool was used to search *Rab3A* upstream region for the 546 Positional Weight Matrices corresponding to vertebrate transcription factors in the TRANSFAC v8.4 [40]. P-value of 0.0002 was used as threshold. If obvious redundancies occurred in which different PWMs for the same TF, lower-scoring overlapping binding sites were manually removed. TFs present in the 16 enriched motifs are highlighted in bold.

(a)

| **Transcription factor binding sites identified in *Rab3A* MCE1** | | | | |
| --- | --- | --- | --- | --- |
| ID | Description | Score | Position | |
| M00919.mat | **E2F** | -10.83 | -1397 | -1387 |
| M00255.mat | GC_box | -9.95 | -1397 | -1384 |
| M00807.mat | EGR | -9.52 | -1396 | -1386 |
| M00716.mat | ZF5 | -8.68 | -1394 | -1387 |
| M00803.mat | **E2F** | -8.70 | -1393 | -1388 |
| M01009.mat | HES1 | -9.10 | -1391 | -1377 |
| M00986.mat | Churchill | -9.03 | -1391 | -1386 |
| M00915.mat | **AP-2** | -8.87 | -1390 | -1378 |
| M00085.mat | ZID | -9.24 | -1389 | -1377 |
| M01001.mat | DEAF1 | -10.14 | -1388 | -1364 |
| M00470.mat | **AP-2gamma** | -8.57 | -1387 | -1379 |
| M00261.mat | **Olf-1** | -9.24 | -1384 | -1363 |
| M00920.mat | **E2F** | -10.36 | -1378 | -1367 |
| M00189.mat | **AP-2** | -9.10 | -1376 | -1365 |
| M00982.mat | KROX | -8.89 | -1376 | -1363 |
| M00244.mat | **NGFI-C** | -12.84 | -1375 | -1364 |
| M00243.mat | Egr-1 | -11.41 | -1375 | -1364 |
| M00803.mat | **E2F** | -8.52 | -1374 | -1369 |
| M00491.mat | MAZR | -8.75 | -1373 | -1361 |
| M00986.mat | Churchill | -9.74 | -1372 | -1367 |
| M00114.mat | **Tax/CREB** | -10.54 | -1371 | -1357 |
| M00378.mat | Pax-4 | -9.37 | -1370 | -1359 |
| M00279.mat | MIF-1 | -9.17 | -1370 | -1353 |
| M00373.mat | Pax-4 | -8.90 | -1369 | -1349 |
| M00333.mat | ZF5 | -9.20 | -1368 | -1356 |
| M00797.mat | HIF-1 | -8.63 | -1367 | -1354 |
| M00953.mat | AR | -9.19 | -1357 | -1331 |
| M00957.mat | PR | -8.57 | -1357 | -1331 |
| M00775.mat | NF-Y | -10.32 | -1333 | -1321 |
| M00740.mat | **Rb:E2F-1:DP-1** | -13.07 | -1331 | -1324 |
| M00982.mat | KROX | -9.64 | -1328 | -1315 |
| M01002.mat | DEAF1 | -8.86 | -1328 | -1304 |
| M00243.mat | Egr-1 | -9.90 | -1327 | -1316 |
| M00652.mat | **Nrf-1** | -9.63 | -1326 | -1317 |
| M00273.mat | R | -9.17 | -1325 | -1305 |
| M00929.mat | **MyoD** | -11.93 | -1324 | -1307 |
| M00695.mat | **ETF** | -8.76 | -1324 | -1318 |
| M00800.mat | **AP-2** | -9.25 | -1322 | -1307 |
| M00532.mat | RP58 | -8.74 | -1322 | -1311 |
| M00002.mat | E47 | -8.74 | -1322 | -1308 |
| M00277.mat | Lmo2_complex | -11.18 | -1321 | -1310 |
| M00176.mat | **AP-4** | -11.32 | -1320 | -1311 |
| M00693.mat | E12 | -9.74 | -1320 | -1310 |
| M00993.mat | TAL1 | -8.91 | -1320 | -1311 |
| M00927.mat | **AP-4** | -12.07 | -1319 | -1311 |
| M00644.mat | LBP-1 | -9.29 | -1319 | -1313 |
| M00804.mat | E2A | -8.54 | -1319 | -1306 |
| M00698.mat | HEB | -10.84 | -1318 | -1311 |
| M00712.mat | **myogenin** | -8.61 | -1318 | -1311 |
| M00644.mat | LBP-1 | -8.57 | -1318 | -1312 |
| M00651.mat | NF-muE1 | -9.69 | -1317 | -1309 |
| M00920.mat | **E2F** | -9.05 | -1317 | -1306 |
| M00083.mat | MZF1 | -9.32 | -1313 | -1306 |
| M00986.mat | Churchill | -8.66 | -1311 | -1306 |
| M00449.mat | Zic2 | -10.18 | -1310 | -1302 |
| M00320.mat | Muscle_TATA_box | -9.62 | -1309 | -1293 |
| M00231.mat | MEF-2 | -9.13 | -1309 | -1288 |
| M00963.mat | T3R | -10.60 | -1289 | -1281 |
| M00236.mat | Arnt | -10.54 | -1289 | -1274 |
| M00771.mat | ETS | -9.56 | -1273 | -1262 |
| M00025.mat | Elk-1 | -11.83 | -1271 | -1258 |
| M00074.mat | c-Ets-1(p54) | -11.69 | -1271 | -1259 |
| M00678.mat | Tel-2 | -10.21 | -1271 | -1262 |
| M00971.mat | Ets | -9.15 | -1269 | -1262 |
| M00634.mat | GCM | -8.58 | -1265 | -1254 |
| M00086.mat | Ik-1 | -9.49 | -1264 | -1252 |
| M00281.mat | RFX1 | -9.21 | -1255 | -1238 |
| M00104.mat | CDP_CR1 | -9.22 | -1252 | -1243 |
| M00077.mat | GATA-3 | -9.45 | -1250 | -1242 |
| M00194.mat | **NF-kappaB** | -8.83 | -1246 | -1233 |
| M00647.mat | **LXR** | -8.82 | -1246 | -1229 |
| M00008.mat | **Sp1** | -9.47 | -1241 | -1232 |
| M00649.mat | MAZ | -8.61 | -1240 | -1233 |
| M00323.mat | Muscle_initiator_sequences-19 | -9.89 | -1236 | -1216 |
| M01028.mat | **NRSF** | -9.17 | -1234 | -1216 |
| M00721.mat | CACCC-binding_factor | -8.57 | -1233 | -1218 |
| M00797.mat | HIF-1 | -9.48 | -1232 | -1219 |

(b)

| **Transcription factor binding sites identified in *Rab3A* MCE2** | | | | |
| --- | --- | --- | --- | --- |
| ID | Description | Score | Position | |
| M00935.mat | NF-AT | -8.92 | -305 | -296 |
| M00209.mat | NF-Y | -8.70 | -304 | -291 |
| M00531.mat | NERF1a | -9.01 | -294 | -277 |
| M00514.mat | ATF4 | -9.08 | -283 | -272 |
| M00764.mat | **HNF-4_direct_repeat_1** | -9.15 | -281 | -269 |
| M00917.mat | **CREB** | -8.68 | -281 | -271 |
| M00998.mat | PBX | -9.63 | -266 | -255 |
| M00150.mat | Brachyury | -8.94 | -262 | -239 |
| M00173.mat | AP-1 | -9.51 | -251 | -241 |
| M00287.mat | NF-Y | -8.63 | -246 | -231 |
| M00238.mat | Barbie_Box | -9.24 | -233 | -219 |
| M00694.mat | E4F1 | -9.09 | -228 | -219 |
| M00706.mat | TFII-I | -8.82 | -227 | -219 |
| M00775.mat | NF-Y | -12.20 | -182 | -170 |
| M00254.mat | CCAAT_box | -11.12 | -181 | -170 |
| M00309.mat | ACAAT | -9.08 | -179 | -171 |
| M00687.mat | alpha-CP1 | -8.69 | -179 | -169 |
| M00302.mat | NF-AT | -9.20 | -175 | -164 |
| M00444.mat | VDR | -8.53 | -159 | -145 |
| M00977.mat | **EBF** | -8.65 | -158 | -148 |
| M00646.mat | LF-A1 | -8.62 | -153 | -146 |
| M00251.mat | XBP-1 | -8.52 | -153 | -137 |
| M00986.mat | Churchill | -9.03 | -149 | -144 |

Table s3. (a) The seven families of related PWMs that we defined as a module to search for additional mouse gene promoters. (b) Mouse genome 5 kb upstream regions were searched based on a 7-motif module derived from Rab3a MCE1 (see main text) thus identifying 42 genes. (c) Expression of these 42 genes were significantly higher than all that of other genes in the genome in the following 18 tissues in the Novartis symAtlas compendium. Wilcoxon rank sum test p-values are shown. Among the 18 tissues, 10 tissues are neural.

**(a)**

| NGFI-C, Egr-2/3, Sp1, KROX |
| --- |
| E2F |
| Lmo2_complex, AREB6, RP58, E12, myogenin, E2A, TAL1, TFE |
| Zic2/3 |
| T3R |
| Tel-2, Ets, ELF-1 |
| CDP_CR1/3 |

**(b)**

| **Genes identified by genome-wide search based on Rab3A MCE1 CRM** | |
| --- | --- |
| IDs | Brief Description |
| NM_009483 | ubiquitously transcribed tetratricopeptide |
| NM_028125 | BTB (POZ) domain containing 4 |
| NM_011958 | origin recognition complex subunit 4 |
| NM_009073 | rod outer segment membrane protein 1 |
| NM_177153 | hypothetical protein LOC320399 |
| NM_025368 | hypothetical protein LOC66124 |
| NM_011328 | secretin |
| NM_023247 | nuclear protein E3-3 |
| NM_199470 | cadherin-like 24 |
| NM_177177 | hypothetical protein LOC320504 |
| NM_030240 | hypothetical protein LOC67278 |
| NM_198651 | hypothetical protein LOC381218 |
| NM_178113 | hypothetical protein LOC78658 |
| NM_016781 | AMP-activated protein kinase, noncatalytic |
| NM_009415 | triosephosphate isomerase 1 |
| NM_010923 | neuronatin isoform alpha |
| NM_008065 | GA repeat binding protein, alpha |
| NM_010268 | ganglioside-induced |
| NM_007425 | advanced glycosylation end product-specific |
| NM_013569 | voltage-gated potassium channel, subfamily H, |
| NM_012048 | polymerase (DNA directed), kappa |
| NM_008420 | potassium voltage gated channel, Shab-related |
| NM_178049 | hypothetical protein LOC217125 |
| NM_180960 | neuronatin isoform beta |
| NM_013627 | paired box gene 6 |
| NM_027356 | hypothetical protein LOC70240 |
| NM_080638 | major vault protein |
| NM_175267 | hypothetical protein LOC77868 |
| NM_028069 | mu-protocadherin |
| NM_026378 | DALR anticodon binding domain containing 3 |
| NM_013604 | metaxin 1 |
| NM_008173 | nuclear receptor subfamily 3, group C, member 1 |
| NM_009410 | topoisomerase (DNA) III alpha |
| NM_030206 | cytoglobin |
| NM_153513 | hypothetical protein LOC229600 |
| NM_029100 | selenoprotein N, 1 |
| NM_153544 | hypothetical protein LOC217216 |
| NM_007726 | cannabinoid receptor 1 (brain) |
| NM_023824 | progestin and adipoQ receptor family member IV |
| NM_183252 | hypothetical protein LOC66297 |
| NM_146025 | hypothetical protein LOC217125 |
| NM_011786 | arachidonate lipoxygenase 3 |
|  |  |

(c)

| **Tissue** | **p-value** |
| --- | --- |
| Cerebral cortex | 3.93E-02 |
| Preoptic | 1.72E-02 |
| Olfactory bulb | 1.95E-02 |
| Substantia nigra | 2.30E-02 |
| Cerebellum | 2.79E-02 |
| Frontal cortex | 2.80E-02 |
| Spinal cord upper | 4.48E-02 |
| Spinal cord lower | 2.98E-02 |
| Dorsal root ganglia | 2.92E-02 |
| Hypothalamus | 3.11E-02 |
| B220+ B-cells | 3.79E-02 |
| Pituitary | 3.11E-02 |
| Thymus | 3.31E-02 |
| Large intestine | 1.18E-02 |
| Snout epidermis | 2.89E-02 |
| Medial olfactory epithelium | 3.27E-02 |
| retina | 3.67E-02 |
| kidney | 3.83E-02 |

**Table s4. List of primer sets used in this study**

| **Primer sets** |  |  |
| --- | --- | --- |
| Construct ID | Forward Primer | Reversed Primer |
| Homology arms A | AAAAGGCGCGCCTGCTTGTTTCGCCTGTTAGCACCA | AAAACCCGGGCTGCGGGAGGCGTGGCACAGA |
| Homology arms B | CGCGTTAATTAAGTGTGTAGGGCTGGAGGGGAGATG | CGCGTTAATTAAGAGTAGGTGGGCTGCTCACCAGTC |
| eGFP-BAC junction 1 | CTCCTCCCAGTGCAGAGAAC | CCGGTGAACAGCTCCTCG |
| eGFP-BAC junction 2 | ATCACTCTCGGCATGGACG | CTGCAGTGACAGCCACAGTT |
| eGFP-1 | ATGGTGAGCAAGGGCGAGGAGC | TTACTTGTACAGCTCGTCCATGCCG |
| eGFP-2 | CCTACGGCGTGCAGTGCTTCAGC | CGGCGAGCTGCACGCTGCGTCCTC |
| BAC-EGFP-5.5kb | AGGGGAAGCAGGAAATAAGC | GGCCTCAGAAAACTTGATGC |
| Rab3a F1 | GCCAGCGTTGTCTCAGCTTAG |  |
| Rab3a F2 | TCCCGCAGATGGCTTCC |  |
| Rab3a F3 | AAACCATCTACCGCAACGAC |  |
| Rab3a R1 |  | GCCTGGTTTCTTGTCAGCTC |
| ST-F/R | GCCAGCGTTGTCTCAGCTTAG | AGCGAGAGTCTGTGGCGG |
| LT-F/R | TCCCGCAGATGGCTTCC | AGGACTCCTTCTGCCCATAGC |
| E3-4-F/R | GGCTGACCACCTGGGCTT | AATGTTGTCCTTGGCGCTG |
| pGL-1 | AGGGGAAGCAGGAAATAAGC | GGCCTCAGAAAACTTGATGC |
| pGL-2 | AGGGGAAGCAGGAAATAAGC | CTGCGGGAGGCGTGGCACAG |
| pGL-3 | AGGGGAAGCAGGAAATAAGC | GGGAGGACACACCACACAC |
| pGL-4 | AGGGGAAGCAGGAAATAAGC | GCGCTCACCTTACCCTCTCT |
| pGL-5 | AGGGGAAGCAGGAAATAAGC | CCTGGTAAGCCACTGGAAGA |
| pGL-6 | AGGGGAAGCAGGAAATAAGC | GTTCTCTGCACTGGGAGGAG |
| pGL-7 | CGTCTTCCAGTGGCTTACCA | CCTGGTAAGCCACTGGAAGA |
| pGL-8 | TCCCCTAACGTCACAGCTTC | CCTGGTAAGCCACTGGAAGA |
| pGL-9 | GCAGGACGTCAGCTAGGACT | CCTGGTAAGCCACTGGAAGA |
| pGL-10 | AGGGGAAGCAGGAAATAAGC | CCAACGCCCTTTACGATATG |
| pGL-11 | AGGGGAAGCAGGAAATAAGC | GGTGGGAATCTGGGGTCTAT |
| pGL-12 | AGGGGAAGCAGGAAATAAGC | CAGGAAGCAGAACTGGGAAC |
| pGL-13 | CTCCTCCCAGTGCAGAGAACA | CCTGGTAAGCCACTGGAAGA |
| pGL-14 | CAAGATGTATGCCTGCCAAT | CCTGGTAAGCCACTGGAAGA |
| pGL-15 | CCTGAGTGCTTCACGTCTCA | CCTGGTAAGCCACTGGAAGA |
| pGL-16 | CCCAGGTCCACACACTGAG | GGGAGGACACACCACACAC |
| pGL-17 | GTCTCGCATTTGGCATTCTT | GGTCACTCGAGCCTCTCCAT |
| pGLc1 | ATCAGGATTGTGCCTGCTG | GGCCTCAGAAAACTTGATGC |
| Luciferase mRNA | TATCCGCTGGAAGATGGAAC | AATCTCACGCAGGCAGTTCT |
| CAMK2N1.3 | CGCAGCACATTTCCTCTCTA | TGTGTTCCCAGCTCAGCTTT |
| CAMK2N1.4 | GAGAAGGGGGTCCTCACATT | CAGGCTGGGGAGAAGTAAAA |
| SNAP25.75_6 | CCTAGTGAGGGCCAAGCATA | GGAGAGGAGCAGAATTTCCTT |
| SNAP25.72 | TAGCCTCCCACTTGGCTATG | GCTGTGTCTGAGTCCCCTCT |
| SV2a1.1 | AAAATGGGGAATAATGTGTTTAAAAG | GCTTTGCCAATGCTCTAGGA |
| Rab3c.28-29 | TGGCCTTATGTAAGTGTTGCAC | TGTTATATGGCAAGGCACGA |
| Scamp5.4 | TGCCACTCTCCACTGAAATG | GTTTTAAGGCATGGGTGTGG |
| Scamp5.7 | ATATAAAATGGGGGCGCTGT | GCTTCTCCTGCTCTCTCCTG |
| Snap25.76 | TCAGTAAGCAAATTGCAGCAG | AGACAGACGGGGAGCTGAG |
| Stxbp1.1 | TCCACCCCATACTTGTCCAT | GACGTGTGGGAATTCAGCAT |
| Scamp5.1 | AGCTGTCCCCACATCCTTTT | TTTTCCTGTGGAGGGAGAAC |
| Camk2G.23 | TCTGGGAAGGGGGTCATAAT | AGTGTGCCTTGCCTCTTCTC |
| NAPG.18 | CCCAGGGCAGGTTTCTTC | GCAAAAGAGCCCTCTCCTCT |
| Camk1G.523 | CTGTTTGTGGGGAGTGAGGT | GTCTGTGGGATGGGAAGC |
| Rims1.394 | GTCAGTTAGGCGGAGTCTCG | CCTTGGGAGAGATCAGTGGA |
| STX18.12 | AGGGTGAGGGGCCAGTATC | GAGACGGGGTGGAGGACT |
| SYT8.4 | CGTCTGCCGTGTGTTCTG | TTTGTGCCTCTCCAGAATGA |
| Camk2G.22 | GAGCTGCTATCCCCAACTCC | ACAGGCAGGCCTATGGGTAA |
| NAPG.7 | CTGAGCAGGCAGATCTGTGA | TGCACACAACTTCCTTGGAT |
| RIMS1.389 | GCCCTGGTTTGTGATAAATTAAA | GGACAGAAACATCCCCACCT |
| Stx18.15 | GATCCGGCCTTATCTGGTTC | TACCTGGCAATCACACTCCA |
| SYT8.2 | AAAGAGGAGGCCTGGGAGT | AATATGCACCCAGAGGTTGG |
| Camk1G.528 | CCACTCTCTCCCTTGTCAGC | ACCAGATTGCTGTGGAAGC |
|  |  |  |
