# Supplementary material for: Motifs and cis-regulatory modules mediating the expression of genes co-expressed in presynaptic neurons
Source: Genome Biol. 2009 Jul 1;10(7):R72. doi: 10.1186/gb-2009-10-7-r72 (PMC2728526; doi:10.1186/gb-2009-10-7-r72)
Supplement: Additional data file 2 — Figure S1 shows Rab3A amplification in cDNA and genomic DNA (gDNA) samples. Figure S2 shows the screen of functional elements in the non-coding region of the Rab3A locus. Figure S3 shows fine mapping of the promoter region of Rab3A. Figure S4 shows the screen of the 1.5 kb upstream region of Rab3A for potential regulatory element(s). Figure S5 shows the screen and characterization of the first intron of Rab3A for regulatory element(s). Figure S6 shows the masking of MCE5 by the presence of MCE1 and MCE2 in Neuro2a cells. Figure S7 shows the statistical evaluation of the scoring strategy for MCEs. [file gb-2009-10-7-r72-S2.pdf]

Additional data file 2

Figure s1. *Rab3A* amplification in cDNA and genomic DNA (gDNA) samples. (a) Schematic representation of *Rab3A* gene and two splicing forms referred to as the long transcript (LT) and the short transcript (ST). The positions of primers used for RT-PCR are represented as arrows. (b) Comparison of PCR amplicons in cDNA and gDNA samples. Lane 1: cDNA; F1/R2. Lane 2: cDNA; F2/R2. Lane 3: gDNA; F1/R2. Lane 4: gDNA; F2/R2. Lane 5: cDNA; F1/R1. Lane 6: cDNA; F2/R1. Lane 7: gDNA; F1/R1. Lane 8: gDNA; F2/R1.

Figure s2. Screen of functional elements in the non-coding region of *Rab3A* locus. (a) Gene structure of *Rab3A* is illustrated. MCEs are represented as red open boxes, coding exons of *Rab3A* as blue blocks and non-coding UTR's as thinner blocks. The 5'UTR of *Rab3A* in ST is shown as an unfilled blue box and designated as +1. (b) A schematic representation of deletion constructs containing different upstream regions fused with pGL *Firefly Luciferase* reporter (green block). (c) *Luciferase* activity measurement of constructs in (b) in Neuro2a cells. Strong negative element(s) was defined in the first 500bp of intronic region and promoter region was defined between MCE2 and MCE4.

Figure s3. Fine mapping of the promoter region of *Rab3A* gene. (a) Gene structure of *Rab3A* is shown and the TSS of the ST is designated as +1. (b) Schematic representation of *Luciferase* constructs containing different length of region between MCE2 and 5'UTR. (c) *Luciferase* activity measurement of constructs in (b) in Neuro2a cells. Core promoter region was defined as in MCE3 region.

Figure s4. Screen of the 1.5 kb upstream region of *Rab3A* gene for potential regulatory element(s). (a) Gene structure of *Rab3A* is illustrated and the TSS of the ST is designated as +1. (b) Schematic representation of a series of *Luciferase* constructs by deleting sequence from the 3' end. (c) Measurement of *Luciferase* activity of constructs in (b) in Neuro2a cells. One enhancer was defined, corresponding to MCE1. (d) Schematic representation of a series of *Luciferase* constructs by deleting sequence from the 5' end. (e) Measurement of *Luciferase*

activity of constructs in (d) in Neuro2a cells. One enhancer was defined, corresponding to MCE2. Potential negative element(s) may exist between MCE1 and MCE2.

Figure s5. Screen and characterization of the first intro of *Rab3A* gene for regulatory element(s). (a) Gene structure of *Rab3A* is illustrated and the TSS of the ST is designated as +1. (b) Schematic representation of a series of *Luciferase* constructs by screening sequence of the first intron. (c) Measurement of *Luciferase* activity of constructs in (b) in Neuro2a, HEK293 and Hela cells. Two potential negative elements were identified. (d) Schematic representation of *Luciferase* constructs under control of SV40 promoter (yellow block) and SV40 enhancer (orange block). pGLc-3 was generated by placing *Rab3A* intronic sequence (+147, +556) upstream to the SV40 promoter. (e) Measurement of *Luciferase* activity of constructs in (d) in Neuro2a cells. The intronic sequence (+147, +556) when placed upstream to the SV40 promoter, fails to reduce *Luciferase* expression.

Figure s6. Mask of MCE5 effect by the presence of MCE1 and MCE2 in Neuro2a cells. (a) Gene structure of *Rab3A* is illustrated and the TSS of the ST is designated as +1. (b) Schematic representation of *Luciferase* constructs testing for MCE5. (c) *Luciferase* activity of constructs in (b) in Neuro2a, HEK293 and Hela cells. In pGLc1, MCE5 leads to decreased *Luciferase* activity in all 3 cell lines. However, in pGL-1, effect of MCE5 seems to be masked by *Rab3A* upstream region in Neuro2a cells. No such mask is observed in HEK293 and Hela cell lines. (d) *Luciferase* mRNA level of each construct was examined by RT-PCR in Neuro2a cells. β-actin gene level is used as control for RNA loading. Changes in mRNA level correspond to changes in *Luciferase* activity.

Figure s7. Statistic evaluation of the scoring strategy for MCEs. (a) Percentage of total MCEs in each expression cluster when score is more than 0. P-value was calculated based on Fisher exact test. (b) Percentage of total MCEs in each expression cluster when score is more than 90. P-value was calculated based on Fisher exact test. (c) Percentage of total MCEs in each cluster when the median's score is more than 0. The median's score represents the median of all MCEs corresponding to a given gene when arranging MCEs by gene. P-value was calculated based on Wilcoxen rank sum test.

Figure s1

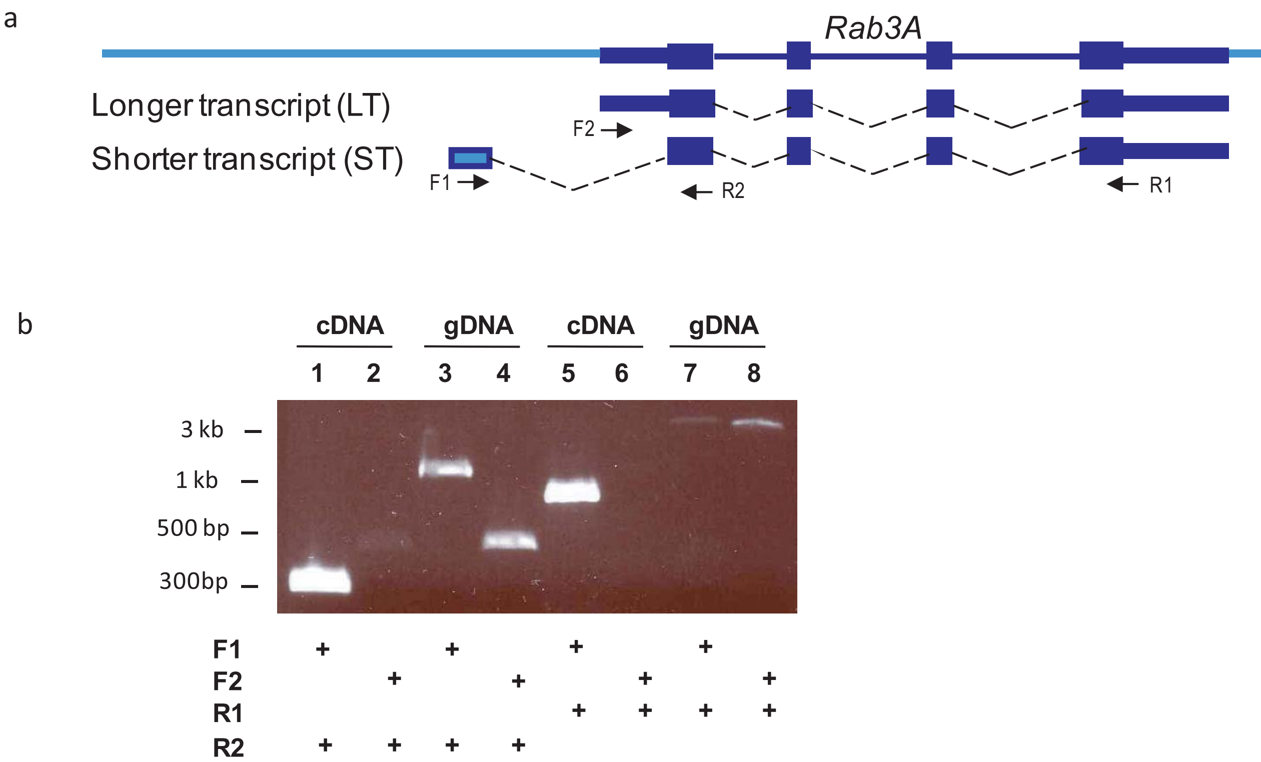
a
Rab3A
Longer transcript (LT)
Shorter transcript (ST)
F2
F1
R2
R1
b
cDNA
gDNA
cDNA
gDNA
1 2 3 4 5 6 7 8
3 kb
1 kb
500 bp
300bp
F1 + + + +
F2 + + + +
R1 + + + +
R2 + + + +

Figure s2.

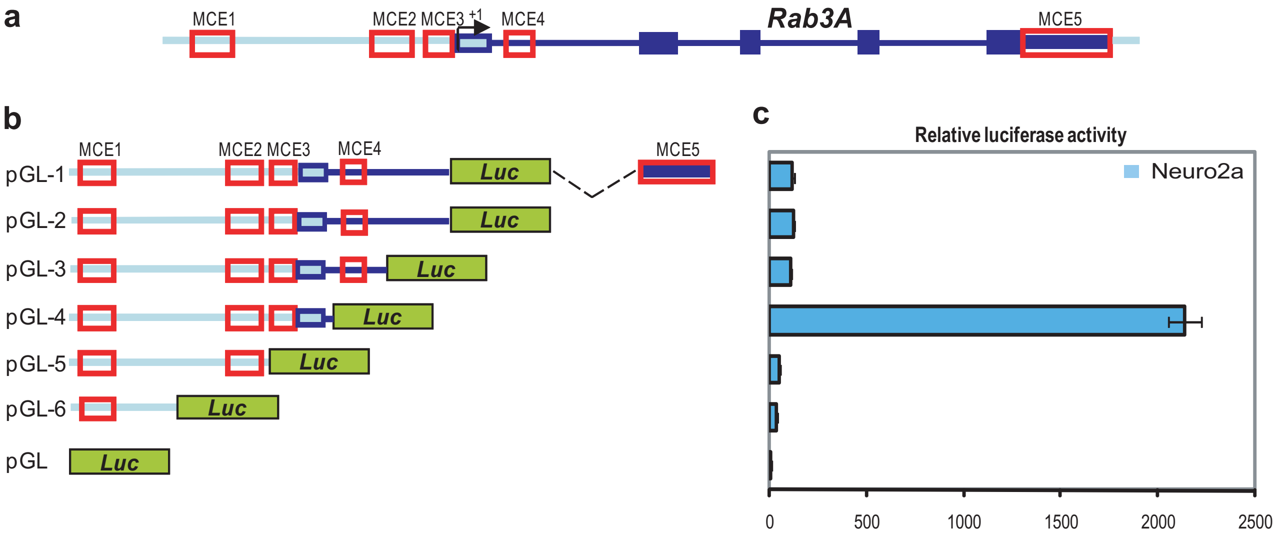
a
MCE1
MCE2
MCE3
+1
MCE4
Rab3A
MCE5
b
MCE1
MCE2
MCE3
MCE4
MCE5
pGL-1
pGL-2
pGL-3
pGL-4
pGL-5
pGL-6
pGL
Luc
c
Relative luciferase activity
Neuro2a
0
500
1000
1500
2000
2500

Figure s3.

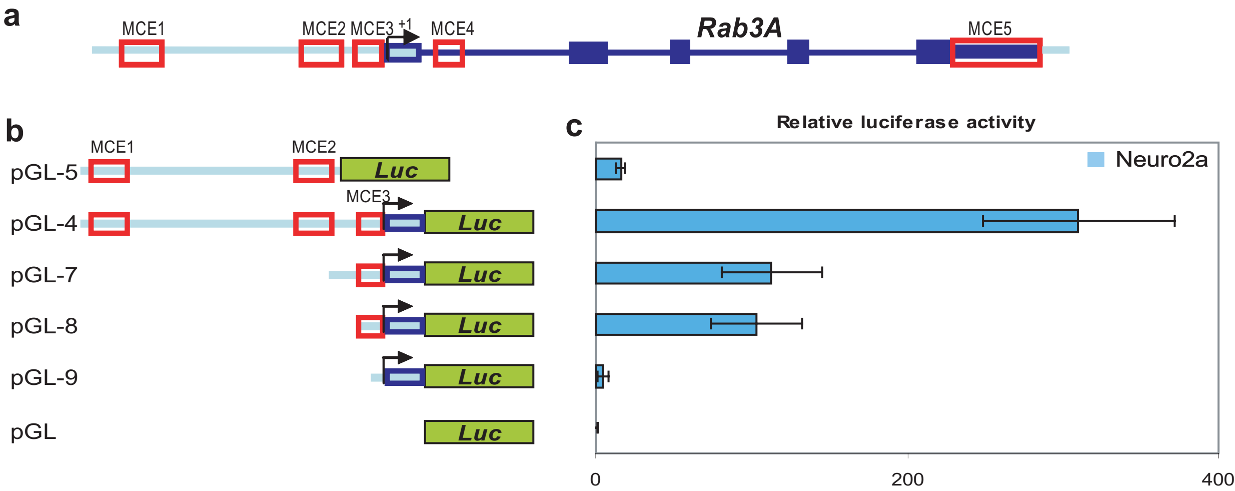

a
MCE1
MCE2
MCE3
+1
MCE4
Rab3A
MCE5
b
MCE1
MCE2
MCE3
pGL-5
pGL-4
pGL-7
pGL-8
pGL-9
pGL
Luc
c
Relative luciferase activity
Neuro2a
0
200
400

Figure s4.

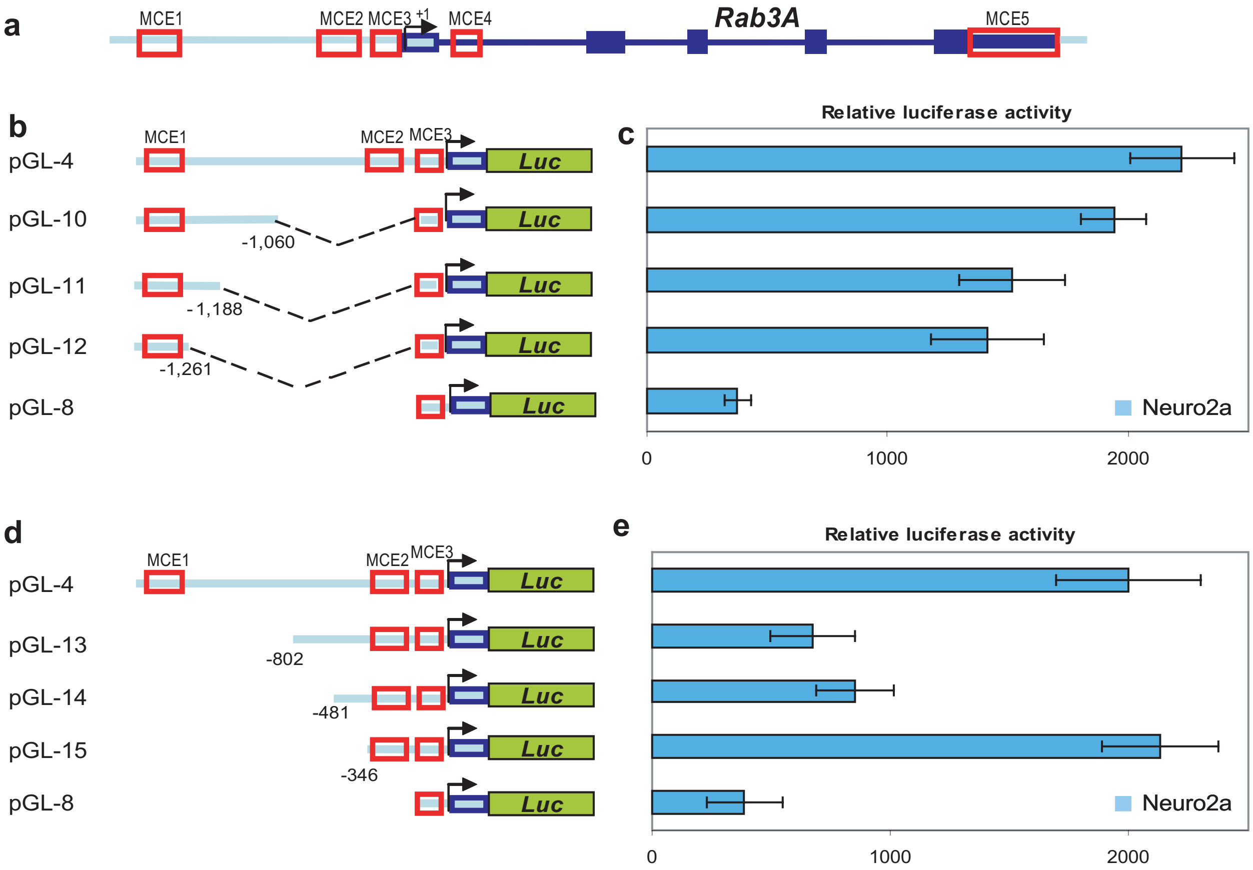
a
MCE1
MCE2
MCE3
+1
MCE4
Rab3A
MCE5
b
MCE1
MCE2
MCE3
pGL-4
pGL-10
pGL-11
pGL-12
pGL-8
Luc
-1,060
- 1,188
-1,261
c
Relative luciferase activity
Neuro2a
0
1000
2000
d
MCE1
MCE2
MCE3
pGL-4
pGL-13
pGL-14
pGL-15
pGL-8
Luc
-802
-481
-346
e
Relative luciferase activity
Neuro2a
0
1000
2000

Figure s5.

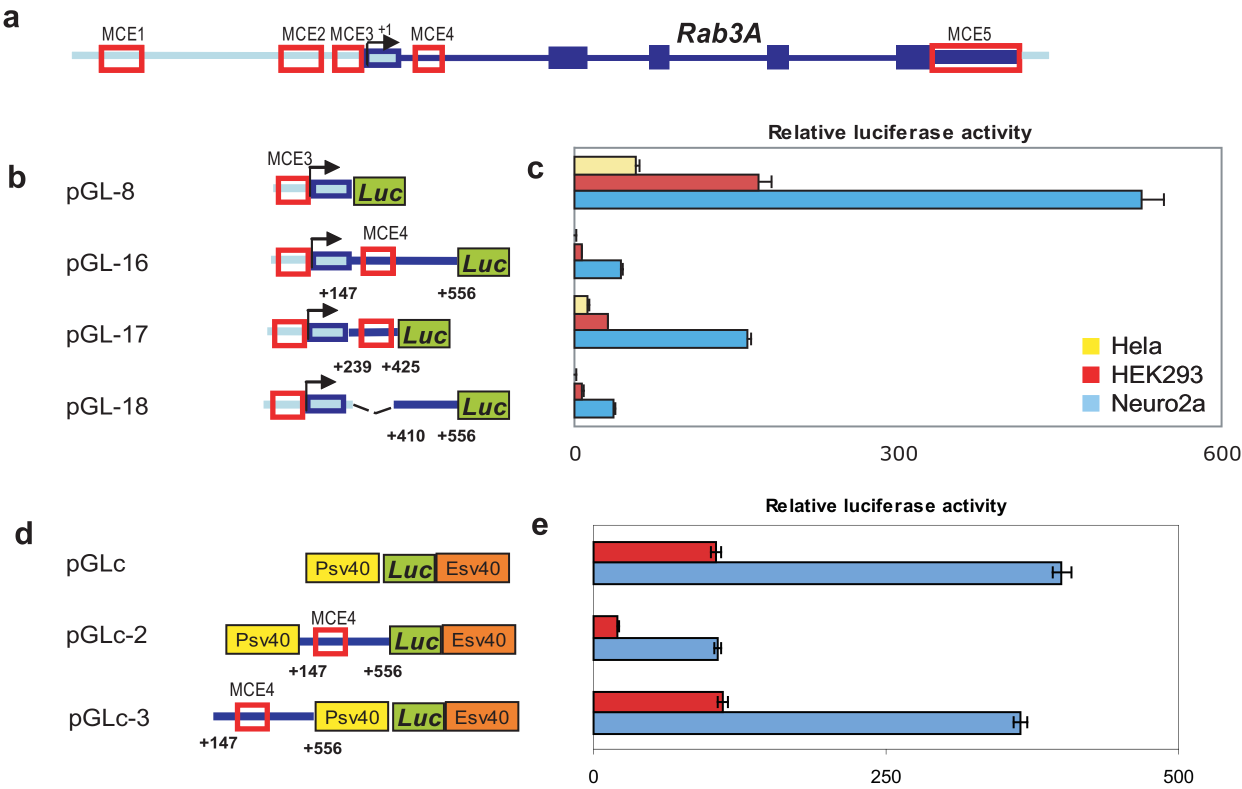

a
MCE1
MCE2
MCE3
+1
MCE4
Rab3A
MCE5
b
pGL-8
pGL-16
pGL-17
pGL-18
MCE3
MCE4
Luc
+147
+556
+239
+425
+410
+556
c
Relative luciferase activity
Hela
HEK293
Neuro2a
0
300
600
d
pGLc
pGLc-2
pGLc-3
Psv40
Luc
Esv40
MCE4
+147
+556
e
Relative luciferase activity
0
250
500

Figure s6.

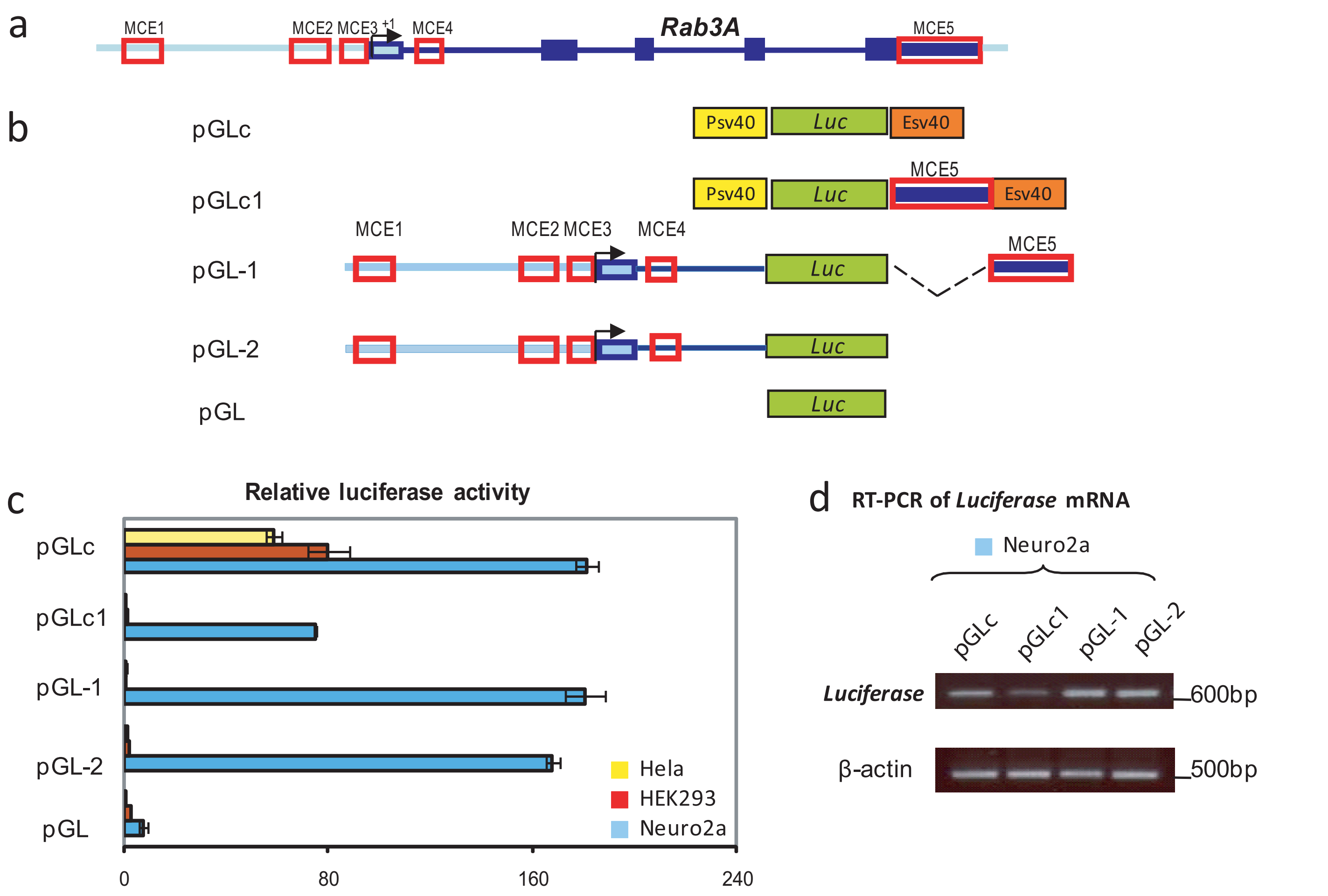
a
MCE1
MCE2
MCE3
+1
MCE4
Rab3A
MCE5
b
pGLc
Psv40
Luc
Esv40
pGLc1
MCE5
pGL-1
MCE1
MCE2 MCE3
MCE4
pGL-2
pGL
c
Relative luciferase activity
Hela
HEK293
Neuro2a
0
80
160
240
d
RT-PCR of Luciferase mRNA
Neuro2a
Luciferase
600bp
β-actin
500bp

Figure s7

a

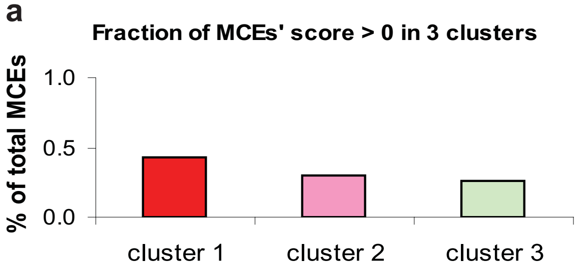

P value
c1 vs c2: 0.011 ; c1 vs c3: 0.0007

b

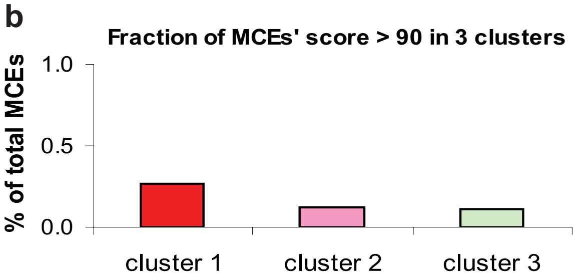

P value
c1 vs c2: 0.0006; c1 vs c3: 0.00002

c

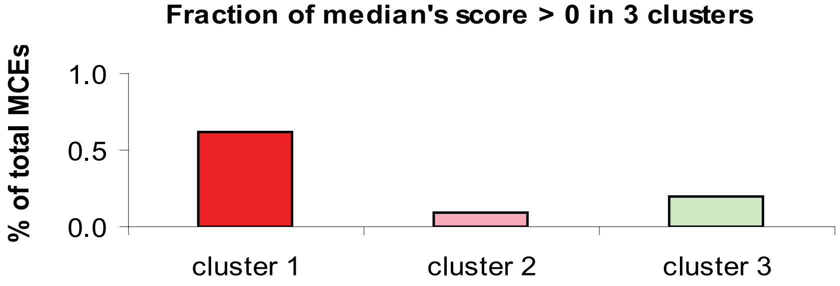

P value
c1 vs c2: 0.005
c1 vs c3: 0.006
